# Insights from the COVID-19 pandemic: trends in development assistance committee countries’ aid allocation, 2011–2021

**DOI:** 10.1080/16549716.2023.2258707

**Published:** 2023-09-21

**Authors:** Shuhei Nomura, Cyrus Ghaznavi, Kazuki Shimizu, Alton Cao, Miho Sassa, Manae Uchibori, Rauniyar Santosh Kumar, Lisa Yamasaki, Hana Tomoi, Haruka Sakamoto

**Affiliations:** a Department of Health Policy and Management, School of Medicine, Keio University, Tokyo, Japan; b Department of Global Health Policy, Graduate School of Medicine, The University of Tokyo, Tokyo, Japan; c Tokyo Foundation for Policy Research, Tokyo, Japan; d Better Co-Being, Tokyo, Japan; e Department of Medicine, University of California San Francisco, San Francisco, USA; f Department of International Medical Education, Graduate School of Medicine, Nagoya University, Nagoya, Japan; g Ocean Policy Research Institute, Sasakawa Peace Foundation, Tokyo, Japan; h Center Hospital of the National Center for Global Health and Medicine, Tokyo, Japan; i Department of Infectious Disease Epidemiology, Faculty of Epidemiology and Population Health, London School of Hygiene and Tropical Medicine, London, UK; j School of Tropical Medicine and Global Health, Nagasaki University, Nagasaki, Japan; k Department of Hygiene and Public Health, Tokyo Women’s Medical University, Tokyo, Japan

**Keywords:** ODA, OECD, DAC, COVID-19, SDGs

## Abstract

**Background:**

Official Development Assistance (ODA) significantly aids sustainable development in low- and middle-income countries (LMICs). However, the COVID-19 pandemic has impacted aid allocation, posing challenges for attaining the Sustainable Development Goals (SDGs).

**Objective:**

This study explores and underscores the profound implications of shifts in ODA allocation by Development Assistance Committee (DAC) member countries, resulting from the COVID-19 pandemic, offering a unique perspective on the evolving landscape of international aid.

**Methods:**

Drawing from the gross ODA disbursement data for LMICs by DAC member countries from 2011 to 2021, a linear regression analysis assessed the changes in ODA amount, ODA-to-gross national income (GNI) ratio, sectoral aid allocation, and the balance between bilateral and multilateral aid, primarily focusing on the differences pre- and post-COVID-19. For non-specialised multilateral agencies’ core funding, the OECD’s methodology for calculating imputed multilateral ODA was employed to estimate ODA flows.

**Results:**

The study found an increasing trend in the total ODA provided by DAC member countries from 2011 to 2021. However, the average ODA/GNI ratio showed a slight but significant decrease before the pandemic, followed by an increase after the COVID-19 pandemic. The health sector received the highest percentage of aid after the pandemic, with a marked increase in both bilateral and multilateral aid. However, other sectors such as humanitarian aid, water and sanitation, and energy experienced a significant decrease in sectoral aid share.

**Conclusions:**

Emerging from this analysis is a strong recommendation for DAC members to re-evaluate aid objectives and escalate their financial commitments to reinforce SDGs and sustainable development efforts. While the rise in health aid is essential, other sectors also require equal focus to offset the ramifications of the COVID-19 pandemic. Understanding the intricacies of aid allocation can improve aid efficacy, culminating in greater, transformative results for recipient countries.

## Introduction

The COVID-19 pandemic has undeniably shaken the core of sustainable development, especially in low- and middle-income countries (LMICs), causing a significant delay in attaining the Sustainable Development Goals (SDGs) [1,2]. These countries, already grappling with resource constraints, found themselves facing an amplified struggle, navigating a myriad of crises. In today’s society, where global challenges intersect across nations, understanding and optimising the role of Official Development Assistance (ODA) becomes imperative [3]. ODA remains an important lifeline for many LMICs in their progress towards SDGs, necessitating the formulation of innovative and robust ODA policies in this new landscape [4].

Since 1970, donor expenditures on ODA have consistently failed to meet the agreed-upon 0.7% of gross national income (GNI) expenditure target in most DAC countries [5,6]. The COVID-19 pandemic has further intensified the funding and resource scarcities experienced by LMICs. Beyond immediate healthcare needs, the pandemic has disrupted economic activities and exhausted government revenue [7]. In 2022, the International Monetary Fund (IMF) projected that low-income countries (LICs) would need an additional 440 billion USD in financing by 2026 to alleviate the pandemic’s impact and revert to their pre-pandemic development trajectories [8]. In the light of this, the paramount role of ODA in extending the much-needed financial bridge for LMICs is evident more than ever before [9].

The international discourse around ODA, particularly in its efficiency and effectiveness, has been rejuvenated due to the pandemic’s pressures [10]. The Organisation for Economic Co-operation and Development (OECD) 2020 report emphasises ODA’s potential as a catalyst in stimulating private and domestic financing in LMICs [11]. Studies like those by Wang et al. have revealed nuanced relationships between ODA, urbanisation, renewable energy development, and technical assistance [12–15]. Such findings, centred on the dynamics and consequences of aid, fortify the case for a more strategic, context-aware deployment of ODA. In this realm, fostering international collaboration and adapting ODA strategies in the light of geopolitical challenges, especially during the COVID-19 era, becomes crucial [16].

Standing distinct from the extant literature, this study seeks to offer an in-depth analysis of ODA policies post-COVID-19. By examining the sectoral allocations of ODA by Development Assistance Committee (DAC) member countries from 2011 to 2021, we introduce a data platform that reflects an evolving ODA paradigm in these unprecedented times. Ultimately, this study not only underscores novel contributions to the discourse, but also aspires to shape ODA strategies, ensuring they resonate with the multifaceted challenges and opportunities awaiting LMICs in the post-pandemic world.

## Methods

### Data

In this study, we utilised data on ODA projects administered by the governments of all 29 DAC countries between 2011 and 2021. We obtained this data from the OECD iLibrary [17], which provides information on gross disbursements of ODA, aid type, and target aid sector for each project and year. Aid type was categorised as bilateral aid or multilateral aid. The aid sectors were classified based on purpose codes, also known as Creditor Reporting System (CRS) codes, which are used by the OECD to classify aid activities.

Based on previous studies [18,19], the following sector categories were used in this study: 110s for education; 120s and 130s for health; 140s for water and sanitation; 151s for government and civil society; 152s for conflict, peace, and security; 160s for other social services; 210s and 220s for infrastructure; 230s for energy; 240s and 250s for financial services and business support; 310s (including forestry and fishing) and 43,040 (rural development) for agriculture; 321s, 322s, and 323s for industry, construction, and mining; 331s for trade policy; 332s for tourism; 410s for environmental protection; 430s, excluding 43,040 (rural development) for multisector; 510s for general budget support; 520s and 530s for food aid and commodity assistance; 600s for debt relief; 720s, 730s, and 740s for humanitarian aid; 910s for donor administration costs; 930s for refugees in donor countries; and 998s for unspecified.

Unless specifically stated otherwise, the current values of ODA for each year were used; however, when converting the current values from 2011 to 2020 to the 2021 constant value, we used the gross domestic product (GDP) deflator from the OECD national accounts. For Lithuania, Hungary, Poland, and the Slovak Republic, data for 2011 to 2013, 2011 to 2013, 2011 to 2012, and 2011 to 2012, respectively, were not available within the OECD iLibrary.

### Estimating imputed multilateral aid by sector

In an improvement over our past approaches, when providing core funding to multilateral agencies that do not specialise in specific aid sectors, we estimated sector-specific ODA using the OECD’s methodology for calculating imputed multilateral ODA [20]. In Step 1, we calculated the percentage of ODA disbursements for each aid sector of each agency based on the reports from multilateral agencies to the OECD (α: sectoral share of the total ODA of the agency) [21]. In Step 2, we multiplied the total ODA of each DAC member country by α for each multilateral agency to estimate country-level sector-specific ODA flows through the agency [22]. The validity of this methodology was confirmed in our previous work [23] and elsewhere [24]. Some multilateral agencies were unable to calculate an α value because their contribution data is not listed within the OECD iLibrary. In our previous studies, we did not account for the contributions to these agencies in our sectoral analysis. However, a major innovation in this study was considering agencies grouped into high-level groups based on the OECD channel catalogue as ‘others’ (e.g. ‘other UN entities’, ‘other multilateral institutions’). They then substituted the aggregated values of other agencies in the same high-level group with available data as their α value.

### Analyses of the sectoral ODA disbursements across the countries

To examine the sectoral trends in ODA disbursements of the 30 DAC countries, three main analyses were conducted. First, the ODA amount and the ODA/GNI ratio from 2011 to 2021 were plotted to identify trends, with separate plots for 2020 and 2021 with and without ODA for COVID-19 response (CRS code = 12264). Secondly, the sectoral proportions were visualised using a heatmap for each year.

To evaluate the disbursement trends and the impact of the COVID-19 pandemic, a linear regression model was developed with a continuous year variable and a binary variable indicating the pre-COVID-19 (2019 or earlier) or post-COVID-19 (2020 or later) periods as non-explanatory variables. This analysis was performed separately for total ODA and by aid type (bilateral and multilateral). The study focused on 30 DAC countries as a whole, using an improved methodological approach. Additional analyses were conducted for each G7 country. Together, these G7 countries represent over 70% of total ODA disbursements, with further analyses for the other remaining DAC member countries.

Third, our methodology introduced an innovative aspect: examining the relationship between per capita ODA for COVID-19 response and COVID-19 impact from 2020 to 2021 (i.e. as of 31 December 2021) in recipient countries of DAC ODA disbursements. The COVID-19 impact was assessed using the reported number of deaths and cases per population as well as excess mortality rates due to the pandemic. In addition, given that ODA disbursements for a given year are largely based on the government budget approved in the previous year, we also conducted an analysis on the relationship between per capita ODA for COVID-19 response in 2021 and the reported COVID-19 mortality in 2020 (i.e. as of 31 December 2020).

Apart from ODA data, GNI statistics were extracted from the World Bank’s World Development Indicators [25,26]. The reported numbers of COVID-19 deaths and cases were obtained from Our World in Data [27]. Statistical significance is defined as *p* < 0.05. Excess mortality rates were downloaded from the Global Health Data Exchange (GHDx) [28].

## Results

**Figure 1.**
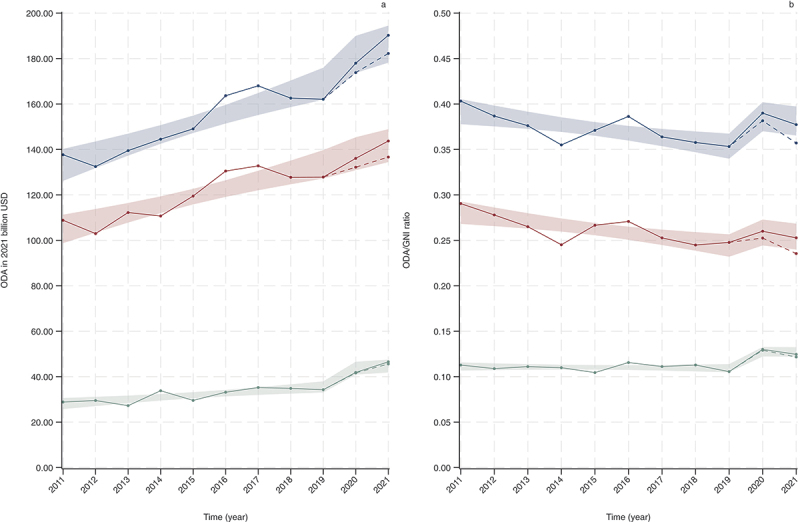
Trend in total ODA (a) in 2021 USD and average ODA/GNI ratio (b) for 30 DAC member countries from 2011 to 2021. the blue line represents total ODA, the red line represents bilateral ODA, and the green line represents multilateral ODA. The dotted line represents ODA not including COVID-19 response. ODA: official development assistance; GNI: gross national income; DAC: development assistance committee. Shading represents the confidence interval bands of linear regression predictions.

Figure 1 illustrates the trend of ODA (in 2021 USD) and the average ODA/GNI ratio for 30 DAC member countries from 2011 to 2021. After adjustment for the pandemic variable, the total, bilateral, and multilateral ODA amounts of the 30 DAC member countries were all found to be statistically significantly increasing annually, with regression coefficients of 4.4670 in 2021 billion USD/year (95% confidence interval 2.7350 to 6.1989), 3.5529 (2.0122 to 5.0935), and 0.9141 (0.3128 to 1.5154), respectively. Furthermore, an increase in multilateral ODA was also observed after the pandemic (7.3381, 2.4078 to 12.2684). The share for the COVID-19 response of the total ODA was 2.34% in 2020 and 4.20% in 2021.

On the other hand, a linear regression analysis revealed a statistically significant decreasing trend in the ODA/GNI ratio each year (−0.0047, −0.0081 to −0.0013) after adjusting for the pandemic variable. However, as indicated by the coefficient of the pandemic variable, the ratio increased after the pandemic compared to before the pandemic (0.0371, 0.0093 to 0.0649). The mean ratio over the 11-year period was 0.3746 (standard deviation 0.0161). Although bilateral aid showed a decreasing trend each year (−0.0045, −0.0075 to −0.0015), there was no significant change after the pandemic. Moreover, there was no yearly change observed in multilateral aid over the 11-year period, although there was an increasing trend after the COVID-19 pandemic (0.0183, 0.0092 to 0.0274). The numerical data for Figure 1 are available in Supplementary Table S1.

**Figure 2.**
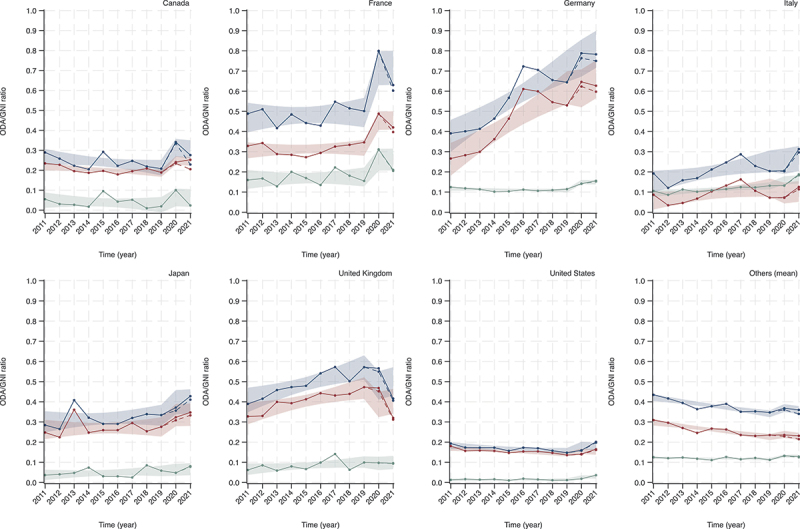
Trend in ODA/GNI ratios for G7 countries and other DAC member countries (average) from 2011 to 2021. the blue line represents total ODA, the red line represents bilateral ODA, and the green line represents multilateral ODA. The dotted line represents ODA not including COVID-19 response. The blue line represents total ODA, the red line represents bilateral ODA, and the green line represents multilateral ODA. The dotted line represents ODA not including COVID-19 response. ODA: official development assistance; GNI: gross national income; DAC: development assistance committee. Shading represents the confidence interval bands of linear regression predictions.

Figure 2 shows the trend in the ODA/GNI ratio for the G7 countries and the average for other DAC member countries from 2011 to 2021. In 2019, before the COVID-19 pandemic, Germany had the highest ODA/GNI ratio at 0.64, followed by the United Kingdom (UK) at 0.57. Only Germany and the UK showed a significant increasing trend in the ODA/GNI ratio each year (0.0432, 0.0237 to 0.0628; 0.0200, 0.0051 to 0.0350). In 2021, Germany still had the highest ODA/GNI ratio at 0.78, and Canada and France showed a significant increase (0.1056, 0.0276 to 0.1837; 0.2162, 0.0697 to 0.3628). Japan had the highest share for the COVID-19 response in ODA among the G7 countries in 2020 (4.09%), while Canada had the highest in 2021 (17.10%). Bilateral aid showed similar results. Although no G7 country, except Italy (0.0046, 0.0003 to 0.0090), showed an annual increase in the ratio of multilateral aid, Germany and the United States demonstrated a significant increase after the pandemic (0.0432, 0.0248 to 0.0616; 0.0138, 0.0007 to 0.0269). The numerical data and regression results for Figure 2 are available in Supplementary Tables S2 and S3, respectively.

Similar results were observed in the trend of ODA amounts (Supplementary Figure S1 and Supplementary Table S4), in which only Germany (2.1869 in 2021 billion USD/year, 1.3807 to 2.9931) and the UK (0.9199, 0.5283 to 1.3115) showed an increase over the years (Supplementary Table S5). Canada (1.7488, 0.4154 to 3.0823) and France (5.4271, 1.6451 to 9.2091) increased their ODA amounts after the pandemic compared to before the pandemic, while the UK decreased ODA (−5.2475, −8.4581 to −2.0370).

**Figure 3.**
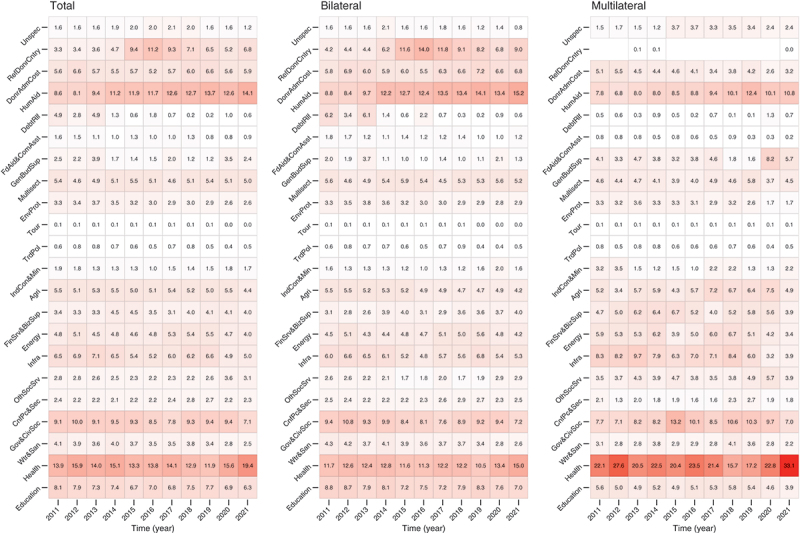
Trend in the overall 22 sectoral ODA shares for 30 DAC member countries from 2011 to 2021. ODA: official development assistance; DAC: development assistance committee.

Figure 3 shows the trend in the proportion of 22 sectors of ODA for all 30 DAC member countries from 2011 to 2021. In 2019, before the COVID-19 pandemic, the humanitarian aid sector had the highest proportion at 13.71%, followed by the health sector at 11.90%. In the regression analysis, the humanitarian aid sector (0.6937%, 0.5133 to 0.8741) and the refugee aid in the donor country (0.7070, 0.0336 to 1.3804) showed an increasing trend in the proportion over the years. Seven other sectors, including debt relief (−0.5745, −0.8834 to −0.2656) and general budget support (−0.2092, −0.4138 to −0.0045), showed a decreasing trend over the years. After the onset of the COVID-19 pandemic, three sectors, including the health sector (5.0861, 1.8594 to 8.3129) and general budget support (2.1310, 0.4530 to 3.8091), showed an increase in proportion, while four sectors, including humanitarian aid (−1.5385, −3.0173 to −0.0596), water and sanitation (−0.7368, −1.1815 to −0.2921), and energy (−1.0741, −1.8490 to −0.2993), showed a decrease, compared to before the pandemic. Following the pandemic in 2021, the health sector constituted the largest proportion of ODA (19.42%). Bilateral aid and multilateral aid showed similar results, with significant increases in health sector aid after the COVID-19 pandemic (2.9143, 1.0549 to 4.7738; 11.0094, 1.9091 to 20.1096). The regression results for Figure 3 are available in Supplementary Table S6.

**Figure 4.**
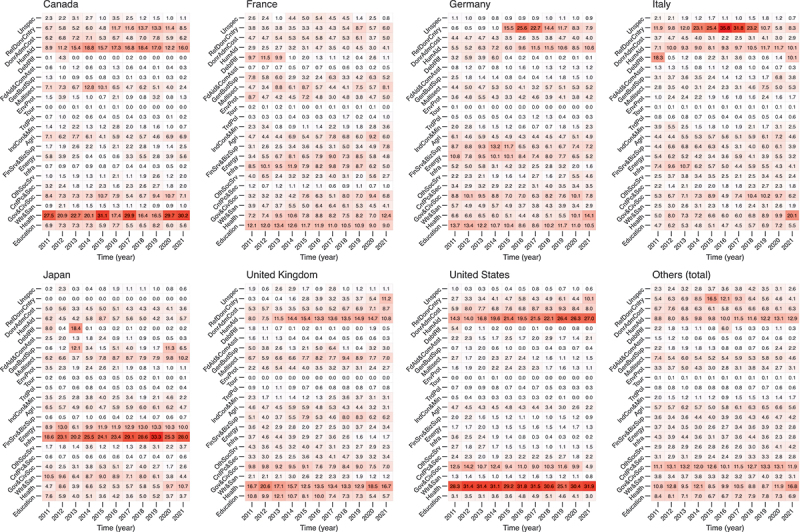
Trend in the 22 sectoral ODA shares for G7 countries and other DAC member countries (total) from 2011 to 2021. ODA: official development assistance; DAC: development assistance committee.

Figure 4 shows the trend in the proportion of 22 sectors of ODA for G7 countries and for other DAC member countries (total) from 2011 to 2021. While the trends in the proportions of the 22 sectors varied among countries, in 2019, before the COVID-19 pandemic, the health sector, government and civil society, and humanitarian aid sectors were among the top five sectors in most of the G7 countries. The humanitarian aid sector showed an increasing trend in all G7 countries except for Japan. After the onset of the COVID-19 pandemic, four G7 countries (Germany, Italy, Japan, and the UK) and other DAC member countries showed an increase in the health sector, while some countries experienced a decrease in other sectors, such as humanitarian aid, water and sanitation, and energy. In 2021 alone, the health sector received the most aid in all G7 countries except for Japan. The regression results for Figure 4 are available in Supplementary Table S7. Similar results were obtained for bilateral (Supplementary Figure S2 and Supplementary Table S8) and multilateral aid (Supplementary Figure S3 and Supplementary Table S9): after the pandemic, many countries experienced an increase in the proportion of their health sector ODA compared to the pandemic, while other sectors experienced significant decreases, though these sectors varied from country to country.

**Figure 5.**
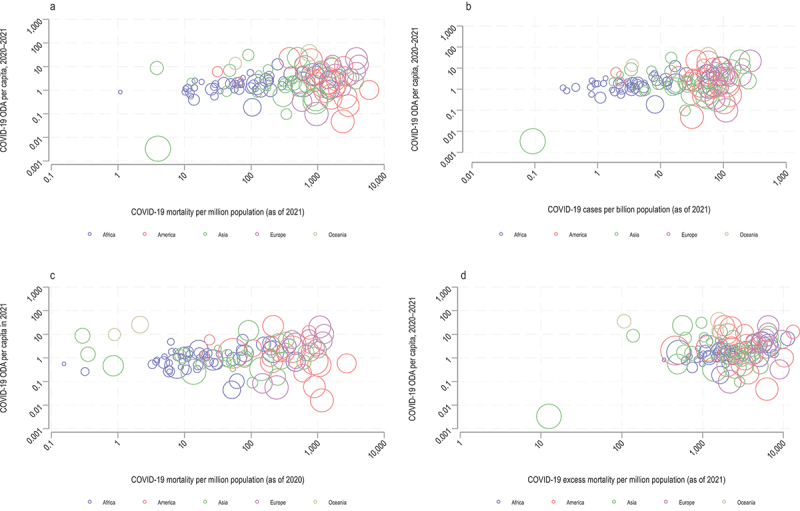
Relationship between the COVID-19 ODA per capita (in 2021 USD) received by ODA recipient countries (a, b, and d for 2020–2021 ODA, and c for 2021) and the COVID-19 impact per population (reported deaths [a as of 2021 and c as of 2020] and cases [b as of 2021], and excess deaths [d as of 2021]). the size of the circle represents each country’s GNI per capita in 2020. ODA: official development assistance; GNI: gross national income; DAC: development assistance committee.

Figure 5 does not show a clear correlation between the COVID-19 ODA per capita (in 2021 USD) received by ODA recipient countries and the COVID-19 impact per population (i.e. reported deaths, reported cases, and excess deaths). The highest correlation coefficient was 0.1297 (*p* = 0.1462), which was between the COVID-19 ODA per capita (2020–2021) received by ODA recipient countries and the COVID-19 deaths per population (as of 2021). Furthermore, there is a large variation in the amount of COVID-19 ODA received by countries with similar GNI levels.

## Discussion

Our analysis showed that the total ODA amount provided by DAC member countries exhibited an increasing trend from 2011 to 2021. Contrary to the findings of Nomura et al. which highlighted the static nature of health investments amidst events like the Ebola outbreak, our study emphasises the more dynamic shifts in ODA priorities, especially concerning health and the COVID-19 pandemic [19]. However, the average ODA/GNI ratio of the member countries showed a slight but significant decreasing trend each year before the COVID-19 pandemic, followed by an increase after the pandemic. After the pandemic, the ratio in the health sector and general budget support increased significantly compared to before the pandemic, while some sectors such as humanitarian aid, water and sanitation, and energy showed an opposite trend significantly.

Economic factors, such as the 2008 global financial crisis and the slow recovery of the world economy, as well as shifts in political priorities for aid from development to national security, trade, and investment, may have contributed to the observed decrease in the ODA/GNI ratio [11,29]. It has been suggested that in some donor countries, ODA is more or less fixed at 0.3% of GNI [30]. However, the COVID-19 pandemic has shown a significant impact on the allocation of aid, particularly in the health sector. This resonates with the findings of Nomura et al. that emphasised the stagnant growth of health aid over the past ten years and the critical need for increased international cooperation in health, especially amidst the COVID-19 pandemic [18]. The COVID-19 pandemic has significantly increased the need for foreign aid in resource-limited settings, as it has caused a rapid and dramatic deterioration of development indicators [31]. In 2020, ODA inflows from DAC member countries to LMICs reached an all-time high, and a considerable portion of the increase in ODA in the health sector was due to donor funds for COVID-19 response efforts [4]. Furthermore, in 2021, we found that health became the sector receiving the most aid in all of the G7 countries except for Japan. Unlike the earlier observation by Nomura et al. that there were no remarkable increases in health sector investments after the Ebola outbreak, our study underscores the transformative effect of the COVID-19 pandemic on aid allocations [19].

The ODA/GNI ratio in bilateral aid showed an annual decreasing trend consistent with the general ODA trend in this study, while a slight increase in this ratio was observed in multilateral aid after the pandemic. In fact, projects by the World Bank, including the International Development Association (IDA), and regional development banks increased their aid by 36.3% from 2019 to 2020 (data not shown in tables/figures). Many development banks frontloaded most of their resources to address the pandemic, which may have resulted in a slowdown in their lending after 2021 if there was no subsequent increase in capital for lending windows or additional resources [32]. In our data, it shows that there was actually a 21.66% decrease in 2021 compared to 2020.

Additionally, a marked increase in the share of health aid was observed in both bilateral and multilateral aid after the COVID-19 pandemic. The increase in the percentage of health aid in bilateral aid suggests a shift towards addressing immediate challenges around COVID-19 in partner countries. Conversely, the increase in aid to the health sector within multilateral aid demonstrates a collaborative endeavour by the international community, exemplified by global pooled fund mechanisms like the COVAX Facility, to tackle the global public health crisis triggered by COVID-19 [31,33]. However, even with the rise in multilateral ODA allocated to the health sector up until 2021, the significant COVID-19-related appeals made by the WHO and the United Nations during that period still faced inadequate funding, resulting in respective shortfalls of 1.8 billion USD and 6.3 billion USD [34]. It is also important to note that the progress towards achieving SDG Goal 3 (health and well-being) has been jeopardised by the COVID-19 pandemic, partly due to the reallocation of aid from other health services towards COVID-19 response efforts. Solely focusing on the overall increase in health aid might overlook the necessity of increasing investment in other essential health services in order to attain the SDGs [35].

Meanwhile, the increase in the percentage of health aid after the pandemic may have come at the expense of other sectors [31]. For example, we found that aid percentages allocated to humanitarian aid, water and sanitation, and energy have decreased after the pandemic compared to before the pandemic. A simple comparison between 2020 and 2021 reveals that although the ODA percentage for the humanitarian aid sector has increased, water and sanitation, as well as energy, have continued to decline. The reallocation of aid funds from other sectors to health has necessitated cuts to existing and planned non-health programmes [36]. Disrupted humanitarian aid is especially concerning for those caught in the midst of conflict [37].

Even prior to the COVID-19 pandemic, progress towards achieving the SDGs was slow [38,39]. According to the SDGs Report 2021, the COVID-19 pandemic has further exacerbated this issue: an additional 119–124 million people have been pushed back into poverty [40]. For the first time since 1998, the global extreme poverty rate has risen, from 8.4% in 2019 to 9.5% in 2020. In the light of these challenges, political leaders face a daunting task of addressing a range of global social issues, including climate change, migration, and refugees [41]. It is important to ensure that the allocation of aid away from specific sectors does not unintentionally hinder partner countries’ progress towards achieving the SDGs [42]. Moreover, as Nomura et al. concluded, such findings should invigorate policy discussions to ensure effective ODA implementation and transparency among the DAC countries [19].

## Implications

Despite a slight increase in the ODA/GNI ratio after the pandemic, the overall decreasing annual trend is still a cause for concern. The decreasing trend in the ODA/GNI ratio may affect the ability of many LMICs to achieve the SDGs. According to the OECD preliminary data for 2022, only a few countries, including Denmark (0.70%), Germany (0.83%), Luxembourg (1.00%), Norway (0.86%), and Sweden (0.90%), have met or exceeded the 0.7% ODA target as a percentage of GNI, calculated on a grant-equivalent basis [43]. DAC members need to re-evaluate aid priorities and increase their financial commitment to development assistance. Innovative financing, such as blended finance and impact investing, can also play a role towards meeting the SDGs [44–46]. It is essential to ensure that aid is effectively targeted and aligned with the development priorities of recipient countries, fostering sustainable and inclusive development [47,48], in accordance with the Paris Declaration and the Accra Agenda for Action [49].

The study findings also reveal considerable variation in aid allocation among donor countries. This observation suggests that multiple factors, including political priorities, economic conditions, and bilateral relationships, influence aid allocation [50–52]. Comprehending these factors can enhance the efficacy and efficiency of aid delivery, ensuring the intended impact is achieved. In relation to the COVID-19 pandemic response, the overall DAC’s allocation of support to recipient countries did not correspond to the pandemic’s consequences on these countries, indicating inefficiencies in aid delivery among donor countries. Therefore, it is crucial to consider these factors when distributing aid to optimise its effectiveness and reach the intended beneficiaries.

Future research in this domain can provide insights into several pertinent aspects. First, understanding the sustainability of increased health-related ODA in the post-pandemic phase will be crucial. This will shed light on whether the focus on health-related assistance was a temporary measure, or indicative of a more lasting shift in aid priorities. Secondly, the effect of political and economic shifts on ODA priorities in the upcoming years should be investigated. Such shifts might have been instigated by the pandemic but could be influenced by other global events in the future. Thirdly, examining the potential long-term implications of aid reallocation on SDGs in recipient countries can offer a broader perspective on developmental objectives. Lastly, exploring how aid allocation patterns might evolve in response to other global challenges, such as climate change or geopolitical tensions, is imperative. Such challenges might reshape the global aid landscape, altering priorities and distribution strategies.

## Limitations

Like previous studies [18,19], this study has limitations due to the nature of ODA. This study used gross disbursements rather than commitments (the amount of aid funding agreed to provide) to analyse ODA. Gross disbursements are subject to instability and are dependent on specific events such as the political or economic instability within a given donor country, as well as the absorptive capacity of the recipient country. Additionally, the regression analysis was conducted with a simple structure for ease of interpretation and due to the scarcity of data points. Therefore, the direction and statistical significance of the coefficients are only valid during the study period (2011–2021).

## Conclusions

In conclusion, our research highlights the necessity for DAC members to reevaluate their aid priorities and augment their financial commitments to bolster the achievement of the SDGs and the sustainable development of recipient countries. The COVID-19 pandemic has profoundly affected aid allocation, especially within the health sector. Although the rise in health aid is vital, other sectors also warrant attention to mitigate the pandemic’s extensive socio-economic ramifications. By comprehending the determinants of aid allocation, we elucidate an important foundation for enhancing the efficacy and efficiency of aid delivery. These findings not only contribute to the academic understanding of aid allocation in the face of unforeseen global challenges, but also have practical implications for policymakers and stakeholders. Potential limitations of our study include our use of gross disbursements over commitments in analysing ODA and the constrained structure of our regression analysis, which binds the significance of our findings to the 2011–2021 period. Future research in this area could focus on understanding the sustainability of increased health-related ODA in the post-pandemic phase, the effect of political and economic shifts on ODA priorities in the coming years, the potential long-term implications of aid reallocation on SDGs in recipient countries, and examining how aid allocation patterns might change in the face of other global challenges, such as climate change or geopolitical tensions.

## Supplementary Material

Supplemental Material

## Paper context

Stronger Official Development Assistance (ODA) policies and their efficient use are critical for achieving the Sustainable Development Goals. We estimated the gross disbursement of ODA of the 29 Development Assistance Committee member countries of the Organisation for Economic Co-operation and Development from 2011 to 2021 by sectors. Our estimates will contribute to the review of the post-COVID-19 pandemic strategic decision-making of future ODA policy discussions.
