# Supplementary material for: Insights from the COVID-19 pandemic: trends in development assistance committee countries’ aid allocation, 2011–2021
Source: Glob Health Action. 2023 Sep 21;16(1):2258707. doi: 10.1080/16549716.2023.2258707 (PMC10515657; doi:10.1080/16549716.2023.2258707)
Supplement: Supplemental Material [file ZGHA_A_2258707_SM2327.docx]

**Supplementary Figure 1: Trend in ODA for 30 DAC member countries from 2011 to 2021.** The blue line represents total ODA, the red line represents bilateral ODA, and the green line represents multilateral ODA. The dotted line represents ODA not including COVID-19 response. ODA: official development assistance; GNI: gross national income; DAC: development assistance committee. Shadows refer to the confidence interval bands of linear regression predictions.

**Supplementary Figure 2: Trend in the 22 sectoral bilateral ODA shares for G7 countries and other DAC member countries (total) from 2011 to 2021.** ODA: official development assistance; DAC: development assistance committee.

**Supplementary Figure 3: Trend in the 22 sectoral multilateral ODA shares for G7 countries and other DAC member countries (total) from 2011 to 2021.** ODA: official development assistance; DAC: development assistance committee.

**Supplementary Table 1: Total ODA (in 2021 USD) and average ODA/GNI ratio for 30 DAC member countries from 2011 to 2021, with the COVID-19 support in parentheses.**

|  | Total (COVID-19) | Bilateral | Multilateral |
| --- | --- | --- | --- |
| Total ODA (in 2021 billion USD) |  |  |  |
| 2011 | 137.6811 | 108.8395 | 28.8416 |
| 2012 | 132.4692 | 102.9584 | 29.5108 |
| 2013 | 139.4910 | 112.2444 | 27.2466 |
| 2014 | 144.5197 | 110.7045 | 33.8153 |
| 2015 | 149.0681 | 119.4977 | 29.5704 |
| 2016 | 163.6555 | 130.4847 | 33.1708 |
| 2017 | 167.9893 | 132.7737 | 35.2157 |
| 2018 | 162.5876 | 127.7319 | 34.8557 |
| 2019 | 162.0887 | 127.8346 | 34.2541 |
| 2020 | 177.9534 (4.1358) | 136.0814 (3.8983) | 41.8720 (0.2374) |
| 2021 | 190.2229 (7.9891) | 143.7012 (7.0630) | 46.5217 (0.9260) |
| Average ODA/GNI ratio |  |  |  |
| 2011 | 0.4032 | 0.2904 | 0.1128 |
| 2012 | 0.3868 | 0.2779 | 0.1089 |
| 2013 | 0.3761 | 0.2651 | 0.1111 |
| 2014 | 0.3550 | 0.2451 | 0.1098 |
| 2015 | 0.3711 | 0.2666 | 0.1045 |
| 2016 | 0.3863 | 0.2708 | 0.1155 |
| 2017 | 0.3638 | 0.2527 | 0.1111 |
| 2018 | 0.3577 | 0.2448 | 0.1129 |
| 2019 | 0.3532 | 0.2477 | 0.1055 |
| 2020 | 0.3900 (0.0084) | 0.2600 (0.0075) | 0.1300 (0.0009) |
| 2021 | 0.3773 (0.0202) | 0.2527 (0.0173) | 0.1246 (0.0029) |

ODA: official development assistance; GNI: gross national income; DAC: development assistance committee.

**Supplementary Table 2: ODA/GNI ratio for G7 countries and other DAC countries, with the COVID-19 support in parentheses.**

|  | Year | 2011 | 2012 | 2013 | 2014 | 2015 | 2016 | 2017 | 2018 | 2019 | 2020 (COVID-19) | 2021 (COVID-19) |
| --- | --- | --- | --- | --- | --- | --- | --- | --- | --- | --- | --- | --- |
| Canada | Total | 0.2891 | 0.2580 | 0.2224 | 0.2045 | 0.2921 | 0.2218 | 0.2468 | 0.2185 | 0.2080 | 0.3412 (0.0071) | 0.2759 (0.0472) |
|  | Bilateral | 0.2343 | 0.2272 | 0.1955 | 0.1871 | 0.1967 | 0.1791 | 0.1949 | 0.2089 | 0.1888 | 0.2408 (0.0070) | 0.2523 (0.0470) |
|  | Multilateral | 0.0548 | 0.0308 | 0.0270 | 0.0174 | 0.0954 | 0.0427 | 0.0519 | 0.0096 | 0.0192 | 0.1004 (0.0001) | 0.0236 (0.0002) |
| France | Total | 0.4887 | 0.5104 | 0.4170 | 0.4849 | 0.4422 | 0.4292 | 0.5478 | 0.5152 | 0.5016 | 0.7999 (0.0016) | 0.6303 (0.0276) |
|  | Bilateral | 0.3289 | 0.3430 | 0.2885 | 0.2847 | 0.2727 | 0.2941 | 0.3258 | 0.3342 | 0.3466 | 0.4884 (0.0002) | 0.4205 (0.0229) |
|  | Multilateral | 0.1598 | 0.1674 | 0.1286 | 0.2002 | 0.1695 | 0.1351 | 0.2220 | 0.1810 | 0.1550 | 0.3116 (0.0014) | 0.2098 (0.0046) |
| Germany | Total | 0.3910 | 0.4013 | 0.4133 | 0.4641 | 0.5670 | 0.7230 | 0.7054 | 0.6554 | 0.6442 | 0.7882 (0.0242) | 0.7829 (0.0328) |
|  | Bilateral | 0.2663 | 0.2828 | 0.2996 | 0.3622 | 0.4637 | 0.6108 | 0.5994 | 0.5458 | 0.5296 | 0.6463 (0.0230) | 0.6271 (0.0296) |
|  | Multilateral | 0.1247 | 0.1184 | 0.1138 | 0.1019 | 0.1033 | 0.1122 | 0.1060 | 0.1095 | 0.1146 | 0.1419 (0.0011) | 0.1558 (0.0033) |
| Italy | Total | 0.1923 | 0.1203 | 0.1587 | 0.1691 | 0.2119 | 0.2470 | 0.2872 | 0.2293 | 0.2041 | 0.2057 (0.0026) | 0.3131 (0.0164) |
|  | Bilateral | 0.0868 | 0.0344 | 0.0459 | 0.0675 | 0.1032 | 0.1324 | 0.1626 | 0.1063 | 0.0723 | 0.0731 (0.0015) | 0.1253 (0.0118) |
|  | Multilateral | 0.1055 | 0.0859 | 0.1127 | 0.1016 | 0.1087 | 0.1147 | 0.1246 | 0.1230 | 0.1317 | 0.1326 (0.0011) | 0.1878 (0.0046) |
| Japan | Total | 0.2845 | 0.2650 | 0.4080 | 0.3214 | 0.2905 | 0.2902 | 0.3200 | 0.3390 | 0.3342 | 0.3713 (0.0152) | 0.4280 (0.0173) |
|  | Bilateral | 0.2478 | 0.2243 | 0.3606 | 0.2475 | 0.2596 | 0.2596 | 0.2949 | 0.2540 | 0.2758 | 0.3233 (0.0149) | 0.3472 (0.0142) |
|  | Multilateral | 0.0367 | 0.0406 | 0.0474 | 0.0739 | 0.0309 | 0.0306 | 0.0251 | 0.0850 | 0.0585 | 0.0479 (0.0003) | 0.0808 (0.0031) |
| United Kingdom | Total | 0.3897 | 0.4152 | 0.4583 | 0.4728 | 0.4792 | 0.5411 | 0.5725 | 0.5022 | 0.5718 | 0.5659 (0.0164) | 0.4159 (0.0100) |
|  | Bilateral | 0.3276 | 0.3299 | 0.3994 | 0.3931 | 0.4127 | 0.4426 | 0.4314 | 0.4390 | 0.4727 | 0.4683 (0.0158) | 0.3202 (0.0082) |
|  | Multilateral | 0.0621 | 0.0853 | 0.0588 | 0.0797 | 0.0665 | 0.0985 | 0.1411 | 0.0631 | 0.0991 | 0.0976 (0.0005) | 0.0958 (0.0018) |
| United States | Total | 0.1933 | 0.1735 | 0.1725 | 0.1728 | 0.1568 | 0.1728 | 0.1697 | 0.1576 | 0.1475 | 0.1594 (0.0021) | 0.2013 (0.0036) |
|  | Bilateral | 0.1804 | 0.1568 | 0.1585 | 0.1566 | 0.1468 | 0.1538 | 0.1545 | 0.1465 | 0.1357 | 0.1412 (0.0020) | 0.1649 (0.0035) |
|  | Multilateral | 0.0128 | 0.0167 | 0.0141 | 0.0161 | 0.0100 | 0.0190 | 0.0152 | 0.0111 | 0.0118 | 0.0182 (0.0000) | 0.0363 (0.0001) |
| Others (mean) | Total | 0.4345 | 0.4164 | 0.3943 | 0.3635 | 0.3780 | 0.3897 | 0.3507 | 0.3527 | 0.3472 | 0.3682 (0.0079) | 0.3597 (0.0197) |
|  | Bilateral | 0.3094 | 0.2962 | 0.2702 | 0.2459 | 0.2670 | 0.2631 | 0.2355 | 0.2309 | 0.2352 | 0.2356 (0.0070) | 0.2315 (0.0166) |
|  | Multilateral | 0.1251 | 0.1203 | 0.1242 | 0.1176 | 0.1109 | 0.1267 | 0.1152 | 0.1219 | 0.1120 | 0.1326 (0.0009) | 0.1282 (0.0031) |

ODA: official development assistance; GNI: gross national income; DAC: development assistance committee.

**Supplementary Table 3: Linear regression coefficients (95% confidence intervals) for the association between year and the COVID-19 pandemic with ODA/GNI ratio for G7 countries and other DAC countries.**

| Total | Year | COVID-19 |
| --- | --- | --- |
| Canada | -0.0068 (-0.0163 to 0.0027) | 0.1056 (0.0276 to 0.1837)* |
| France | 0.0031 (-0.0148 to 0.0210) | 0.2162 (0.0697 to 0.3628)** |
| Germany | 0.0432 (0.0237 to 0.0628)** | -0.0038 (-0.1639 to 0.1563) |
| Italy | 0.0126 (-0.0007 to 0.0259) | -0.0122 (-0.1209 to 0.0966) |
| Japan | 0.0040 (-0.0088 to 0.0168) | 0.0606 (-0.0443 to 0.1656) |
| United Kingdom | 0.0200 (0.0051 to 0.0350)* | -0.1083 (-0.2309 to 0.0142) |
| United States | -0.0036 (-0.0076 to 0.0005) | 0.0314 (-0.0019 to 0.0647) |
| Others | -0.0100 (-0.0141 to -0.0059)*** | 0.0382 (0.0046 to 0.0717)* |
| Bilateral |  |  |
| Canada | -0.0040 (-0.0085 to 0.0006) | 0.0670 (0.0298 to 0.1042)** |
| France | 0.0016 (-0.0081 to 0.0112) | 0.1327 (0.0538 to 0.2116)** |
| Germany | 0.0443 (0.0243 to 0.0643)** | -0.0470 (-0.2110 to 0.1170) |
| Italy | 0.0080 (-0.0030 to 0.0189) | -0.0348 (-0.1245 to 0.0549) |
| Japan | 0.0015 (-0.0102 to 0.0133) | 0.0574 (-0.0389 to 0.1538) |
| United Kingdom | 0.0156 (0.0024 to 0.0288)* | -0.0972 (-0.2054 to 0.0110) |
| United States | -0.0035 (-0.0063 to -0.0006)* | 0.0176 (-0.0057 to 0.0409) |
| Others | -0.0090 (-0.0127 to -0.0054)*** | 0.0218 (-0.0082 to 0.0518) |
| Multilateral |  |  |
| Canada | -0.0028 (-0.0122 to 0.0066) | 0.0386 (-0.0383 to 0.1156) |
| France | 0.0015 (-0.0100 to 0.0130) | 0.0835 (-0.0108 to 0.1778) |
| Germany | -0.0011 (-0.0033 to 0.0012) | 0.0432 (0.0248 to 0.0616)** |
| Italy | 0.0046 (0.0003 to 0.0090)* | 0.0226 (-0.0132 to 0.0585) |
| Japan | 0.0025 (-0.0038 to 0.0088) | 0.0032 (-0.0485 to 0.0549) |
| United Kingdom | 0.0044 (-0.0026 to 0.0113) | -0.0111 (-0.0683 to 0.0461) |
| United States | -0.0001 (-0.0017 to 0.0015) | 0.0138 (0.0007 to 0.0269)* |
| Others | -0.0010 (-0.0025 to 0.0006) | 0.0164 (0.0038 to 0.0290)* |

ODA: official development assistance; GNI: gross national income; DAC: development assistance committee. * p<0.05; ** p<0.01; *** p<0.001.

**Supplementary Table 4: ODA amount (in 2021 billion USD) for G7 countries and other DAC countries, with the COVID-19 support in parentheses.**

|  | Year | 2011 | 2012 | 2013 | 2014 | 2015 | 2016 | 2017 | 2018 | 2019 | 2020 (COVID-19) | 2021 (COVID-19) |
| --- | --- | --- | --- | --- | --- | --- | --- | --- | --- | --- | --- | --- |
| Canada | Total | 4.8335 | 4.3937 | 3.8874 | 3.6721 | 5.2831 | 4.0664 | 4.6602 | 4.2170 | 4.0985 | 6.3800 (0.1326) | 5.4460 (0.9314) |
|  | Bilateral | 3.9176 | 3.8694 | 3.4162 | 3.3601 | 3.5577 | 3.2840 | 3.6804 | 4.0311 | 3.7203 | 4.5020 (0.1316) | 4.9799 (0.9285) |
|  | Multilateral | 0.9158 | 0.5243 | 0.4712 | 0.3120 | 1.7253 | 0.7824 | 0.9798 | 0.1859 | 0.3782 | 1.8780 (0.0010) | 0.4661 (0.0030) |
| France | Total | 13.5363 | 14.0955 | 11.5984 | 13.6172 | 12.5432 | 12.3104 | 16.1154 | 15.4227 | 15.2574 | 22.4148 (0.0458) | 19.1931 (0.8399) |
|  | Bilateral | 9.1100 | 9.4732 | 8.0222 | 7.9946 | 7.7359 | 8.4348 | 9.5837 | 10.0038 | 10.5421 | 13.6844 (0.0052) | 12.8050 (0.6988) |
|  | Multilateral | 4.4263 | 4.6223 | 3.5763 | 5.6225 | 4.8073 | 3.8756 | 6.5317 | 5.4189 | 4.7153 | 8.7304 (0.0405) | 6.3881 (0.1411) |
| Germany | Total | 15.3844 | 15.8069 | 16.3531 | 18.7290 | 23.2592 | 30.4011 | 30.4629 | 28.8219 | 28.6287 | 33.7471 (1.0343) | 34.5347 (1.4477) |
|  | Bilateral | 10.4781 | 11.1408 | 11.8512 | 14.6186 | 19.0230 | 25.6844 | 25.8859 | 24.0048 | 23.5358 | 27.6718 (0.9855) | 27.6626 (1.3038) |
|  | Multilateral | 4.9063 | 4.6662 | 4.5019 | 4.1104 | 4.2362 | 4.7167 | 4.5770 | 4.8171 | 5.0929 | 6.0752 (0.0488) | 6.8721 (0.1439) |
| Italy | Total | 4.1772 | 2.5361 | 3.2828 | 3.5077 | 4.3954 | 5.2443 | 6.2228 | 5.0341 | 4.4892 | 4.1594 (0.0529) | 6.7178 (0.3522) |
|  | Bilateral | 1.8853 | 0.7255 | 0.9506 | 1.4004 | 2.1401 | 2.8100 | 3.5236 | 2.3338 | 1.5912 | 1.4773 (0.0303) | 2.6892 (0.2527) |
|  | Multilateral | 2.2919 | 1.8105 | 2.3321 | 2.1073 | 2.2553 | 2.4343 | 2.6992 | 2.7003 | 2.8981 | 2.6820 (0.0226) | 4.0286 (0.0995) |
| Japan | Total | 13.7738 | 13.0059 | 20.5587 | 16.2705 | 14.9771 | 15.0131 | 16.8694 | 17.9949 | 17.7175 | 18.7452 (0.7655) | 21.9516 (0.8883) |
|  | Bilateral | 11.9964 | 11.0122 | 18.1710 | 12.5292 | 13.3817 | 13.4289 | 15.5469 | 13.4831 | 14.6171 | 16.3246 (0.7528) | 17.8066 (0.7293) |
|  | Multilateral | 1.7774 | 1.9937 | 2.3877 | 3.7413 | 1.5955 | 1.5842 | 1.3226 | 4.5118 | 3.1004 | 2.4206 (0.0127) | 4.1450 (0.1590) |
| United Kingdom | Total | 10.9424 | 11.6244 | 12.9813 | 13.7709 | 14.2698 | 16.4385 | 18.0255 | 16.0039 | 18.7830 | 16.1762 (0.4677) | 12.9673 (0.3112) |
|  | Bilateral | 9.1997 | 9.2354 | 11.3147 | 11.4504 | 12.2900 | 13.4465 | 13.5828 | 13.9923 | 15.5262 | 13.3851 (0.4520) | 9.9815 (0.2561) |
|  | Multilateral | 1.7427 | 2.3890 | 1.6666 | 2.3205 | 1.9798 | 2.9920 | 4.4428 | 2.0116 | 3.2568 | 2.7911 (0.0157) | 2.9858 (0.0551) |
| United States | Total | 36.8788 | 34.1919 | 34.4452 | 35.5448 | 33.0333 | 36.7369 | 37.0227 | 35.3538 | 33.7928 | 35.5947 (0.4619) | 47.5359 (0.8456) |
|  | Bilateral | 34.4338 | 30.9093 | 31.6366 | 32.2253 | 30.9340 | 32.7010 | 33.6992 | 32.8533 | 31.0935 | 31.5223 (0.4537) | 38.9531 (0.8299) |
|  | Multilateral | 2.4450 | 3.2826 | 2.8086 | 3.3195 | 2.0993 | 4.0359 | 3.3235 | 2.5005 | 2.6994 | 4.0724 (0.0081) | 8.5828 (0.0157) |
| Others (total) | Total | 38.1546 | 36.8148 | 36.3841 | 39.4075 | 41.3069 | 43.4447 | 38.6103 | 39.7393 | 39.3216 | 40.7361 (1.1752) | 41.8764 (2.3728) |
|  | Bilateral | 27.8184 | 26.5926 | 26.8819 | 27.1259 | 30.4353 | 30.6951 | 27.2712 | 27.0298 | 27.2085 | 27.5138 (1.0871) | 28.8233 (2.0640) |
|  | Multilateral | 10.3362 | 10.2221 | 9.5022 | 12.2816 | 10.8716 | 12.7496 | 11.3391 | 12.7095 | 12.1131 | 13.2223 (0.0881) | 13.0532 (0.3087) |

ODA: official development assistance; GNI: gross national income; DAC: development assistance committee.

**Supplementary Table 5: Linear regression coefficients (95% confidence intervals) for the association between year and the COVID-19 pandemic with ODA amount (in 2021 USD) for G7 countries and other DAC countries.**

| Total | Year | COVID-19 |
| --- | --- | --- |
| Canada | -0.0330 (-0.1957 to 0.1296) | 1.7488 (0.4154 to 3.0823)* |
| France | 0.2807 (-0.1806 to 0.7420) | 5.4271 (1.6451 to 9.2091)* |
| Germany | 2.1869 (1.3807 to 2.9931)*** | -0.9812 (-7.5914 to 5.6290) |
| Italy | 0.2915 (-0.0066 to 0.5896) | -0.4860 (-2.9301 to 1.9582) |
| Japan | 0.3919 (-0.2694 to 1.0532) | 1.9507 (-3.4710 to 7.3725) |
| United Kingdom | 0.9199 (0.5283 to 1.3115)** | -5.2475 (-8.4581 to -2.0370)** |
| United States | 0.0572 (-0.9261 to 1.0405) | 6.0286 (-2.0333 to 14.0904) |
| Others | 0.3719 (-0.2034 to 0.9473) | 0.0180 (-4.6992 to 4.7353) |
| Bilateral |  |  |
| Canada | 0.0064 (-0.0790 to 0.0918) | 1.0572 (0.3571 to 1.7574)** |
| France | 0.1726 (-0.0932 to 0.4384) | 3.3063 (1.1272 to 5.4855)** |
| Germany | 2.1480 (1.3311 to 2.9649)*** | -2.6159 (-9.3134 to 4.0817) |
| Italy | 0.1787 (-0.0588 to 0.4161) | -0.8284 (-2.7751 to 1.1183) |
| Japan | 0.2362 (-0.3714 to 0.8438) | 1.9705 (-3.0111 to 6.9521) |
| United Kingdom | 0.7340 (0.4013 to 1.0667)** | -4.5801 (-7.3082 to -1.8520)** |
| United States | 0.0130 (-0.6522 to 0.6782) | 2.8899 (-2.5639 to 8.3437) |
| Others | 0.0640 (-0.4028 to 0.5308) | -0.0791 (-3.9062 to 3.7480) |
| Multilateral |  |  |
| Canada | -0.0394 (-0.2112 to 0.1324) | 0.6916 (-0.7172 to 2.1004) |
| France | 0.1081 (-0.1994 to 0.4156) | 2.1208 (-0.4006 to 4.6422) |
| Germany | 0.0389 (-0.0661 to 0.1440) | 1.6347 (0.7734 to 2.4960)** |
| Italy | 0.1129 (0.0062 to 0.2195)* | 0.3424 (-0.5318 to 1.2166) |
| Japan | 0.1557 (-0.1720 to 0.4835) | -0.0197 (-2.7069 to 2.6674) |
| United Kingdom | 0.1859 (-0.0322 to 0.4040) | -0.6674 (-2.4556 to 1.1207) |
| United States | 0.0442 (-0.3321 to 0.4204) | 3.1387 (0.0540 to 6.2233)* |
| Others | 0.3079 (0.0625 to 0.5533)* | 0.0971 (-1.9147 to 2.1089) |

ODA: official development assistance; DAC: development assistance committee. * p<0.05; ** p<0.01; *** p<0.001.

**Supplementary Table 6: Linear regression coefficients (95% confidence intervals) for the association between year and the COVID-19 pandemic with ODA/GNI ratio for all DAC countries**

| Total | Year | COVID-19 |
| --- | --- | --- |
| Education | -0.0774 (-0.2123 to 0.0575) | -0.3534 (-1.4594 to 0.7526) |
| Health | -0.2706 (-0.6642 to 0.1230) | 5.0861 (1.8594 to 8.3129)** |
| Water and sanitation | -0.0620 (-0.1163 to -0.0078)* | -0.7368 (-1.1815 to -0.2921)** |
| Government and civil society | -0.0861 (-0.3308 to 0.1587) | -0.3341 (-2.3410 to 1.6728) |
| Conflict, peace and security | 0.0535 (-0.0041 to 0.1111) | -0.3778 (-0.8500 to 0.0944) |
| Other social services | -0.0643 (-0.1249 to -0.0037)* | 1.2250 (0.7280 to 1.7221)*** |
| Infrastructure | -0.0894 (-0.2676 to 0.0888) | -0.8692 (-2.3302 to 0.5918) |
| Energy | 0.0796 (-0.0149 to 0.1742) | -1.0741 (-1.8490 to -0.2993)* |
| Financial services and business support | 0.0513 (-0.1013 to 0.2039) | -0.0035 (-1.2549 to 1.2478) |
| Agriculture | -0.0340 (-0.1281 to 0.0601) | -0.0777 (-0.8493 to 0.6939) |
| Industry, construction and mining | -0.0482 (-0.1234 to 0.0269) | 0.5949 (-0.0213 to 1.2110) |
| Trade policy | -0.0120 (-0.0464 to 0.0224) | -0.1470 (-0.4292 to 0.1352) |
| Tourism | -0.0056 (-0.0099 to -0.0013)* | -0.0240 (-0.0594 to 0.0115) |
| Environmental protection | -0.0856 (-0.1376 to -0.0336)** | -0.1643 (-0.5906 to 0.2620) |
| Multisector | 0.0213 (-0.0812 to 0.1237) | -0.0973 (-0.9371 to 0.7425) |
| General budget support | -0.2092 (-0.4138 to -0.0045)* | 2.1310 (0.4530 to 3.8091)* |
| Food aid and commodity assistance | -0.0616 (-0.1136 to -0.0096)* | 0.0245 (-0.4019 to 0.4510) |
| Debt relief | -0.5745 (-0.8834 to -0.2656)** | 2.0201 (-0.5123 to 4.5525) |
| Humanitarian aid | 0.6937 (0.5133 to 0.8741)*** | -1.5385 (-3.0173 to -0.0596)* |
| Donor administration costs | 0.0329 (-0.1004 to 0.1662) | -0.2434 (-1.3362 to 0.8494) |
| Refugees in donor country | 0.7070 (0.0336 to 1.3804)* | -4.3990 (-9.9199 to 1.1220) |
| Unspecified | 0.0410 (-0.0278 to 0.1098) | -0.6416 (-1.2057 to -0.0775)* |
| Bilateral |  |  |
| Education | -0.1111 (-0.2688 to 0.0466) | -0.0022 (-1.2949 to 1.2905) |
| Health | -0.1180 (-0.3448 to 0.1088) | 2.9143 (1.0549 to 4.7738)** |
| Water and sanitation | -0.1001 (-0.1430 to -0.0572)** | -0.6144 (-0.9662 to -0.2627)** |
| Government and civil society | -0.2110 (-0.4908 to 0.0687) | 0.3749 (-1.9188 to 2.6685) |
| Conflict, peace and security | 0.0467 (-0.0142 to 0.1077) | -0.3291 (-0.8290 to 0.1709) |
| Other social services | -0.0938 (-0.1550 to -0.0326)** | 1.3418 (0.8401 to 1.8434)*** |
| Infrastructure | -0.0494 (-0.2436 to 0.1447) | -0.2874 (-1.8791 to 1.3043) |
| Energy | 0.0992 (-0.0034 to 0.2017) | -0.8778 (-1.7184 to -0.0372)* |
| Financial services and business support | 0.0748 (-0.0638 to 0.2134) | 0.1080 (-1.0285 to 1.2445) |
| Agriculture | -0.1145 (-0.1652 to -0.0638)** | 0.0773 (-0.3386 to 0.4931) |
| Industry, construction and mining | -0.0159 (-0.0802 to 0.0485) | 0.5577 (0.0300 to 1.0854)* |
| Trade policy | -0.0118 (-0.0547 to 0.0311) | -0.1528 (-0.5045 to 0.1988) |
| Tourism | -0.0095 (-0.0149 to -0.0041)** | -0.0159 (-0.0602 to 0.0285) |
| Environmental protection | -0.0892 (-0.1476 to -0.0308)** | 0.1215 (-0.3571 to 0.6001) |
| Multisector | -0.0013 (-0.1385 to 0.1359) | 0.1949 (-0.9298 to 1.3197) |
| General budget support | -0.1921 (-0.4152 to 0.0309) | 1.1845 (-0.6441 to 3.0130) |
| Food aid and commodity assistance | -0.0672 (-0.1240 to -0.0103)* | 0.1114 (-0.3546 to 0.5773) |
| Debt relief | -0.7165 (-1.1131 to -0.3200)** | 2.3418 (-0.9095 to 5.5932) |
| Humanitarian aid | 0.7355 (0.4972 to 0.9738)*** | -1.4395 (-3.3933 to 0.5143) |
| Donor administration costs | 0.0889 (-0.0619 to 0.2396) | -0.0357 (-1.2716 to 1.2002) |
| Refugees in donor country | 0.8903 (0.0617 to 1.7189)* | -5.2350 (-12.0288 to 1.5587) |
| Unspecified | -0.0340 (-0.1179 to 0.0500) | -0.3383 (-1.0268 to 0.3502) |
| Multilateral |  |  |
| Education | 0.0340 (-0.0716 to 0.1396) | -1.1988 (-2.0645 to -0.3330)* |
| Health | -0.7812 (-1.8911 to 0.3287) | 11.0094 (1.9091 to 20.1096)* |
| Water and sanitation | 0.0746 (-0.0622 to 0.2113) | -1.1288 (-2.2499 to -0.0076)* |
| Government and civil society | 0.3671 (-0.1494 to 0.8835) | -2.9994 (-7.2335 to 1.2346) |
| Conflict, peace and security | 0.0745 (-0.0343 to 0.1833) | -0.4701 (-1.3621 to 0.4219) |
| Other social services | 0.0521 (-0.1470 to 0.2511) | 0.5131 (-1.1191 to 2.1453) |
| Infrastructure | -0.2346 (-0.5220 to 0.0529) | -2.8269 (-5.1837 to -0.4702)* |
| Energy | 0.0104 (-0.2412 to 0.2620) | -1.7012 (-3.7640 to 0.3615) |
| Financial services and business support | -0.0272 (-0.3179 to 0.2636) | -0.5723 (-2.9561 to 1.8116) |
| Agriculture | 0.2610 (-0.0695 to 0.5915) | -0.8271 (-3.5371 to 1.8830) |
| Industry, construction and mining | -0.1635 (-0.4009 to 0.0740) | 0.7059 (-1.2410 to 2.6527) |
| Trade policy | -0.0134 (-0.0432 to 0.0165) | -0.1290 (-0.3737 to 0.1158) |
| Tourism | 0.0084 (0.0028 to 0.0141)** | -0.0603 (-0.1067 to -0.0139)* |
| Environmental protection | -0.0728 (-0.1261 to -0.0194)* | -1.0791 (-1.5164 to -0.6418)*** |
| Multisector | 0.1005 (-0.0594 to 0.2603) | -1.0039 (-2.3144 to 0.3067) |
| General budget support | -0.2594 (-0.5661 to 0.0473) | 4.9315 (2.4169 to 7.4462)** |
| Food aid and commodity assistance | -0.0446 (-0.0958 to 0.0066) | -0.1740 (-0.5938 to 0.2458) |
| Debt relief | -0.0492 (-0.1306 to 0.0322) | 0.8303 (0.1629 to 1.4977)* |
| Humanitarian aid | 0.5274 (0.3118 to 0.7430)*** | -1.3353 (-3.1026 to 0.4321) |
| Donor administration costs | -0.1801 (-0.3007 to -0.0595)** | -0.5072 (-1.4961 to 0.4817) |
| Refugees in donor country | NA | NA |
| Unspecified | 0.3189 (0.1141 to 0.5238)** | -1.9795 (-3.6589 to -0.3001)* |

* p<0.05; ** p<0.01; *** p<0.001. NA: The regression model could not be constructed due to a small number of contribution years

**Supplementary Table 7: Linear regression coefficients (95% confidence intervals) for the association between year and the COVID-19 pandemic with sector shares in G7 countries and other DAC countries**

| Donor | Sector | Year | COVID-19 |
| --- | --- | --- | --- |
| Canada | Education | -0.0158 (-0.2664 to 0.2349) | -1.0832 (-3.1386 to 0.9721) |
|  | Health | -0.7439 (-2.3273 to 0.8396) | 11.4940 (-1.4886 to 24.4767) |
|  | Water and sanitation | -0.0599 (-0.1637 to 0.0439) | -0.0664 (-0.9173 to 0.7845) |
|  | Government and civil society | -0.0472 (-0.5741 to 0.4797) | 1.3472 (-2.9726 to 5.6670) |
|  | Conflict, peace and security | 0.0028 (-0.1762 to 0.1818) | -0.3552 (-1.8227 to 1.1124) |
|  | Other social services | 0.1178 (-0.0219 to 0.2575) | -0.6581 (-1.8037 to 0.4874) |
|  | Infrastructure | -0.0663 (-0.1093 to -0.0232)** | 0.0874 (-0.2655 to 0.4403) |
|  | Energy | -0.0763 (-0.6897 to 0.5370) | 2.3716 (-2.6572 to 7.4004) |
|  | Financial services and business support | 0.0517 (-0.0972 to 0.2007) | -0.4317 (-1.6532 to 0.7899) |
|  | Agriculture | -0.3069 (-0.5756 to -0.0381)* | 2.8320 (0.6283 to 5.0357)* |
|  | Industry, construction and mining | 0.0555 (-0.0991 to 0.2101) | -1.1978 (-2.4653 to 0.0697) |
|  | Trade policy | -0.0623 (-0.1191 to -0.0054)* | 0.0168 (-0.4492 to 0.4828) |
|  | Tourism | -0.0012 (-0.0142 to 0.0118) | -0.0214 (-0.1278 to 0.0851) |
|  | Environmental protection | -0.0392 (-0.3773 to 0.2989) | -0.4744 (-3.2464 to 2.2976) |
|  | Multisector | -0.3780 (-1.0784 to 0.3224) | -2.1120 (-7.8545 to 3.6306) |
|  | General budget support | -0.1424 (-0.1917 to -0.0931)*** | 0.4323 (0.0281 to 0.8364)* |
|  | Food aid and commodity assistance | -0.0645 (-0.1663 to 0.0373) | 0.0207 (-0.8139 to 0.8552) |
|  | Debt relief | -0.2104 (-0.5924 to 0.1715) | 0.6485 (-2.4832 to 3.7802) |
|  | Humanitarian aid | 0.9439 (0.2683 to 1.6195)* | -6.6399 (-12.1791 to -1.1007)* |
|  | Donor administration costs | 0.0362 (-0.2449 to 0.3174) | -0.6869 (-2.9922 to 1.6184) |
|  | Refugees in donor country | 1.1140 (0.4506 to 1.7774)** | -4.9205 (-10.3597 to 0.5187) |
|  | Unspecified | -0.1079 (-0.3421 to 0.1263) | -0.6030 (-2.5231 to 1.3170) |
| France | Education | -0.2433 (-0.4189 to -0.0677)* | -1.4616 (-2.9014 to -0.0218)* |
|  | Health | 0.0470 (-0.4692 to 0.5632) | 1.0962 (-3.1361 to 5.3285) |
|  | Water and sanitation | 0.2663 (0.0548 to 0.4778)* | -2.3626 (-4.0966 to -0.6286)* |
|  | Government and civil society | 0.5630 (0.1575 to 0.9684)* | -0.3047 (-3.6289 to 3.0195) |
|  | Conflict, peace and security | -0.0095 (-0.0750 to 0.0560) | -0.1428 (-0.6799 to 0.3943) |
|  | Other social services | -0.2891 (-0.6822 to 0.1040) | 2.3235 (-0.8995 to 5.5466) |
|  | Infrastructure | -0.1525 (-0.5251 to 0.2200) | -2.7597 (-5.8146 to 0.2951) |
|  | Energy | 0.4889 (0.1650 to 0.8129)** | -4.3864 (-7.0428 to -1.7301)** |
|  | Financial services and business support | 0.1981 (-0.0870 to 0.4831) | 1.8105 (-0.5264 to 4.1474) |
|  | Agriculture | 0.2693 (-0.0994 to 0.6379) | 0.3353 (-2.6870 to 3.3577) |
|  | Industry, construction and mining | -0.0497 (-0.2958 to 0.1964) | 1.7290 (-0.2886 to 3.7467) |
|  | Trade policy | 0.0683 (-0.0661 to 0.2026) | -0.1358 (-1.2375 to 0.9658) |
|  | Tourism | -0.0098 (-0.0273 to 0.0078) | -0.0134 (-0.1572 to 0.1304) |
|  | Environmental protection | -0.3488 (-0.7929 to 0.0953) | 1.6693 (-1.9719 to 5.3106) |
|  | Multisector | 0.0844 (-0.5360 to 0.7047) | 0.5141 (-4.5721 to 5.6003) |
|  | General budget support | -0.3679 (-0.8407 to 0.1048) | 3.1211 (-0.7549 to 6.9971) |
|  | Food aid and commodity assistance | -0.0322 (-0.0712 to 0.0067) | -0.0805 (-0.4000 to 0.2389) |
|  | Debt relief | -1.4179 (-2.1704 to -0.6654)** | 5.4473 (-0.7226 to 11.6171) |
|  | Humanitarian aid | 0.2341 (0.0838 to 0.3844)** | -1.1964 (-2.4286 to 0.0358) |
|  | Donor administration costs | 0.1166 (-0.0339 to 0.2671) | -1.5321 (-2.7660 to -0.2981)* |
|  | Refugees in donor country | 0.4445 (0.1029 to 0.7861)* | -1.0948 (-3.8955 to 1.7059) |
|  | Unspecified | 0.1405 (-0.3492 to 0.6302) | -2.5754 (-6.5900 to 1.4393) |
| Germany | Education | -0.4497 (-0.8617 to -0.0376)* | 2.1752 (-1.2032 to 5.5536) |
|  | Health | -0.1937 (-0.5421 to 0.1547) | 7.5238 (4.6671 to 10.3805)*** |
|  | Water and sanitation | -0.2293 (-0.3705 to -0.0880)** | -0.2768 (-1.4347 to 0.8812) |
|  | Government and civil society | -0.3251 (-0.6436 to -0.0066)* | 2.5780 (-0.0335 to 5.1895) |
|  | Conflict, peace and security | 0.0439 (-0.1196 to 0.2074) | 0.1684 (-1.1721 to 1.5090) |
|  | Other social services | 0.0012 (-0.1496 to 0.1520) | 2.9710 (1.7345 to 4.2074)** |
|  | Infrastructure | -0.3506 (-0.5598 to -0.1414)** | -0.1862 (-1.9017 to 1.5292) |
|  | Energy | -0.3015 (-0.5991 to -0.0040)* | -1.3178 (-3.7573 to 1.1216) |
|  | Financial services and business support | -0.4744 (-1.1118 to 0.1630) | 1.2882 (-3.9377 to 6.5141) |
|  | Agriculture | -0.1527 (-0.3039 to -0.0015)* | 0.9687 (-0.2709 to 2.2084) |
|  | Industry, construction and mining | -0.1555 (-0.3353 to 0.0244) | 1.2767 (-0.1980 to 2.7513) |
|  | Trade policy | 0.0536 (-0.0482 to 0.1555) | -0.6073 (-1.4426 to 0.2279) |
|  | Tourism | -0.0052 (-0.0097 to -0.0007)* | 0.0145 (-0.0226 to 0.0515) |
|  | Environmental protection | -0.0430 (-0.2319 to 0.1459) | -0.0176 (-1.5665 to 1.5314) |
|  | Multisector | 0.0165 (-0.2066 to 0.2396) | 0.4300 (-1.3996 to 2.2595) |
|  | General budget support | -0.1202 (-0.2965 to 0.0561) | 0.6277 (-0.8176 to 2.0729) |
|  | Food aid and commodity assistance | 0.0509 (-0.0264 to 0.1281) | -0.0972 (-0.7303 to 0.5359) |
|  | Debt relief | -0.6981 (-1.1885 to -0.2077)* | 1.6897 (-2.3307 to 5.7101) |
|  | Humanitarian aid | 0.8737 (0.5334 to 1.2140)*** | -3.4206 (-6.2109 to -0.6303)* |
|  | Donor administration costs | -0.0895 (-0.2914 to 0.1123) | 0.4623 (-1.1926 to 2.1173) |
|  | Refugees in donor country | 2.5500 (0.4347 to 4.6652)* | -16.2244 (-33.5672 to 1.1184) |
|  | Unspecified | -0.0013 (-0.0393 to 0.0367) | -0.0261 (-0.3373 to 0.2851) |
| Italy | Education | 0.0817 (-0.1793 to 0.3426) | 0.5753 (-1.5642 to 2.7149) |
|  | Health | 0.2209 (-0.5950 to 1.0367) | 6.9028 (0.2137 to 13.5919)* |
|  | Water and sanitation | -0.0528 (-0.2183 to 0.1128) | 0.0335 (-1.3236 to 1.3906) |
|  | Government and civil society | 0.4505 (-0.0630 to 0.9639) | -2.1121 (-6.3216 to 2.0973) |
|  | Conflict, peace and security | 0.0614 (-0.0449 to 0.1676) | -0.2990 (-1.1702 to 0.5721) |
|  | Other social services | -0.0909 (-0.3226 to 0.1407) | 0.9027 (-0.9965 to 2.8019) |
|  | Infrastructure | -0.5404 (-0.9922 to -0.0886)* | 0.4784 (-3.2259 to 4.1827) |
|  | Energy | -0.2111 (-0.5143 to 0.0921) | 0.8181 (-1.6678 to 3.3039) |
|  | Financial services and business support | -0.1940 (-0.5903 to 0.2023) | 0.7041 (-2.5452 to 3.9535) |
|  | Agriculture | 0.1555 (-0.0754 to 0.3864) | -0.3053 (-2.1984 to 1.5878) |
|  | Industry, construction and mining | -0.3409 (-0.6504 to -0.0314)* | 2.2552 (-0.2824 to 4.7928) |
|  | Trade policy | -0.0408 (-0.0779 to -0.0038)* | 0.1622 (-0.1415 to 0.4658) |
|  | Tourism | 0.0034 (-0.0042 to 0.0110) | -0.0503 (-0.1127 to 0.0121) |
|  | Environmental protection | 0.0259 (-0.1116 to 0.1635) | -0.4463 (-1.5741 to 0.6816) |
|  | Multisector | 0.3297 (-0.0666 to 0.7260) | -0.3741 (-3.6235 to 2.8753) |
|  | General budget support | -0.3501 (-0.6066 to -0.0936)* | 4.7853 (2.6822 to 6.8885)** |
|  | Food aid and commodity assistance | -0.0860 (-0.1561 to -0.0159)* | -0.0830 (-0.6578 to 0.4918) |
|  | Debt relief | -0.9853 (-2.6138 to 0.6432) | 7.7597 (-5.5925 to 21.1120) |
|  | Humanitarian aid | 0.3324 (0.0216 to 0.6432)* | -0.3555 (-2.9037 to 2.1927) |
|  | Donor administration costs | -0.1540 (-0.3835 to 0.0756) | 0.2656 (-1.6164 to 2.1476) |
|  | Refugees in donor country | 1.4736 (-1.1538 to 4.1010) | -21.4873 (-43.0290 to 0.0543) |
|  | Unspecified | -0.0886 (-0.1932 to 0.0160) | -0.1300 (-0.9873 to 0.7274) |
| Japan | Education | -0.2310 (-0.5548 to 0.0929) | 0.0710 (-2.5842 to 2.7263) |
|  | Health | -0.0282 (-0.4251 to 0.3686) | 4.6550 (1.4012 to 7.9088)* |
|  | Water and sanitation | -0.3354 (-0.6783 to 0.0076) | -2.3116 (-5.1235 to 0.5002) |
|  | Government and civil society | 0.0040 (-0.3058 to 0.3138) | -1.5114 (-4.0516 to 1.0288) |
|  | Conflict, peace and security | -0.0173 (-0.0440 to 0.0094) | 0.1845 (-0.0343 to 0.4032) |
|  | Other social services | 0.1095 (-0.1659 to 0.3848) | 0.3478 (-1.9095 to 2.6051) |
|  | Infrastructure | 1.5298 (0.9363 to 2.1233)*** | -6.8500 (-11.7159 to -1.9841)* |
|  | Energy | 0.5412 (-0.0359 to 1.1182) | -4.0409 (-8.7722 to 0.6904) |
|  | Financial services and business support | 0.0330 (-0.0697 to 0.1357) | -0.2246 (-1.0666 to 0.6174) |
|  | Agriculture | 0.0120 (-0.1902 to 0.2142) | -0.3899 (-2.0480 to 1.2681) |
|  | Industry, construction and mining | -0.1445 (-0.4051 to 0.1160) | 1.2148 (-0.9215 to 3.3512) |
|  | Trade policy | -0.0364 (-0.0782 to 0.0053) | -0.0169 (-0.3589 to 0.3251) |
|  | Tourism | -0.0154 (-0.0687 to 0.0379) | -0.1905 (-0.6275 to 0.2466) |
|  | Environmental protection | -0.2250 (-0.3549 to -0.0950)** | 0.1788 (-0.8863 to 1.2440) |
|  | Multisector | 0.3881 (0.0276 to 0.7485)* | 0.7969 (-2.1581 to 3.7518) |
|  | General budget support | -0.1665 (-1.2615 to 0.9284) | 6.3299 (-2.6477 to 15.3076) |
|  | Food aid and commodity assistance | -0.2074 (-0.3314 to -0.0834)** | 0.0257 (-0.9907 to 1.0422) |
|  | Debt relief | -1.1415 (-2.7634 to 0.4805) | 3.3874 (-9.9109 to 16.6857) |
|  | Humanitarian aid | -0.0385 (-0.4788 to 0.4017) | -0.7419 (-4.3515 to 2.8678) |
|  | Donor administration costs | -0.0694 (-0.2603 to 0.1215) | -0.4027 (-1.9679 to 1.1624) |
|  | Refugees in donor country | -0.0003 (-0.0005 to -0.0002)** | 0.0007 (-0.0008 to 0.0021) |
|  | Unspecified | 0.0393 (-0.1662 to 0.2448) | -0.5122 (-2.1972 to 1.1729) |
| United Kingdom | Education | -0.5281 (-0.8473 to -0.2089)** | -0.8220 (-3.4391 to 1.7951) |
|  | Health | -0.7831 (-1.2635 to -0.3027)** | 6.8070 (2.8682 to 10.7458)** |
|  | Water and sanitation | -0.0147 (-0.1047 to 0.0754) | -0.9933 (-1.7316 to -0.2551)* |
|  | Government and civil society | -0.2048 (-0.3778 to -0.0318)* | -0.5310 (-1.9495 to 0.8876) |
|  | Conflict, peace and security | 0.2048 (0.0521 to 0.3575)* | -1.3683 (-2.6203 to -0.1163)* |
|  | Other social services | -0.1934 (-0.3879 to 0.0011) | 0.0238 (-1.5708 to 1.6183) |
|  | Infrastructure | -0.2832 (-0.4538 to -0.1127)** | -0.1905 (-1.5889 to 1.2079) |
|  | Energy | -0.0580 (-0.2636 to 0.1475) | 0.2058 (-1.4795 to 1.8911) |
|  | Financial services and business support | 0.4412 (0.0501 to 0.8324)* | -2.0256 (-5.2326 to 1.1813) |
|  | Agriculture | -0.0227 (-0.2078 to 0.1624) | -1.6566 (-3.1742 to -0.1389)* |
|  | Industry, construction and mining | 0.2313 (-0.0002 to 0.4629) | -0.2445 (-2.1427 to 1.6537) |
|  | Trade policy | -0.0283 (-0.0671 to 0.0105) | 0.0592 (-0.2588 to 0.3771) |
|  | Tourism | -0.0008 (-0.0037 to 0.0021) | -0.0086 (-0.0322 to 0.0150) |
|  | Environmental protection | -0.0678 (-0.3450 to 0.2094) | -0.8389 (-3.1115 to 1.4338) |
|  | Multisector | 0.3767 (0.2072 to 0.5462)** | -2.2006 (-3.5903 to -0.8109)** |
|  | General budget support | -0.2564 (-0.8369 to 0.3241) | 1.4483 (-3.3112 to 6.2078) |
|  | Food aid and commodity assistance | -0.1310 (-0.2298 to -0.0321)* | 0.4112 (-0.3993 to 1.2217) |
|  | Debt relief | -0.2030 (-0.3338 to -0.0722)** | 1.8577 (0.7852 to 2.9302)** |
|  | Humanitarian aid | 0.7748 (0.1221 to 1.4274)* | -3.9763 (-9.3274 to 1.3749) |
|  | Donor administration costs | 0.2424 (-0.0651 to 0.5500) | 1.3490 (-1.1725 to 3.8705) |
|  | Refugees in donor country | 0.5564 (0.1306 to 0.9823)* | 3.0155 (-0.4761 to 6.5071) |
|  | Unspecified | -0.0524 (-0.3055 to 0.2008) | -0.3213 (-2.3965 to 1.7540) |
| United States | Education | 0.2466 (0.0994 to 0.3938)** | -1.8614 (-3.0683 to -0.6545)** |
|  | Health | -0.2217 (-0.8516 to 0.4082) | 2.3019 (-2.8623 to 7.4660) |
|  | Water and sanitation | -0.0086 (-0.0700 to 0.0529) | -0.3344 (-0.8386 to 0.1697) |
|  | Government and civil society | -0.3836 (-0.9104 to 0.1432) | -1.7478 (-6.0670 to 2.5713) |
|  | Conflict, peace and security | 0.0470 (-0.0189 to 0.1128) | -0.5221 (-1.0619 to 0.0176) |
|  | Other social services | -0.1954 (-0.2962 to -0.0946)** | 0.7969 (-0.0294 to 1.6232) |
|  | Infrastructure | -0.4123 (-0.5089 to -0.3157)*** | 0.9618 (0.1697 to 1.7540)* |
|  | Energy | -0.0437 (-0.1021 to 0.0147) | 0.1171 (-0.3618 to 0.5961) |
|  | Financial services and business support | -0.1246 (-0.2084 to -0.0408)** | 0.2886 (-0.3985 to 0.9758) |
|  | Agriculture | -0.1711 (-0.2915 to -0.0507)* | -0.8571 (-1.8442 to 0.1300) |
|  | Industry, construction and mining | -0.0387 (-0.0528 to -0.0246)*** | -0.0411 (-0.1566 to 0.0743) |
|  | Trade policy | -0.0586 (-0.0989 to -0.0183)* | -0.0052 (-0.3356 to 0.3253) |
|  | Tourism | -0.0055 (-0.0151 to 0.0042) | 0.0059 (-0.0733 to 0.0850) |
|  | Environmental protection | 0.0013 (-0.0959 to 0.0985) | -0.8376 (-1.6342 to -0.0409)* |
|  | Multisector | -0.1378 (-0.2849 to 0.0092) | 0.1479 (-1.0577 to 1.3536) |
|  | General budget support | -0.1396 (-0.2807 to 0.0015) | 0.5637 (-0.5930 to 1.7205) |
|  | Food aid and commodity assistance | 0.0222 (-0.1219 to 0.1663) | -0.4178 (-1.5994 to 0.7638) |
|  | Debt relief | -0.4065 (-0.8100 to -0.0030)* | 1.8461 (-1.4625 to 5.1547) |
|  | Humanitarian aid | 1.3595 (0.9492 to 1.7698)*** | -0.3503 (-3.7145 to 3.0139) |
|  | Donor administration costs | 0.2689 (0.0441 to 0.4937)* | -0.9475 (-2.7906 to 0.8957) |
|  | Refugees in donor country | 0.4043 (-0.0199 to 0.8286) | 0.6276 (-2.8511 to 4.1063) |
|  | Unspecified | -0.0021 (-0.0757 to 0.0714) | 0.2647 (-0.3383 to 0.8677) |
| Others | Education | -0.0566 (-0.2385 to 0.1253) | -0.0062 (-1.4977 to 1.4853) |
|  | Health | -0.3093 (-0.8354 to 0.2169) | 5.8618 (1.5477 to 10.1758)* |
|  | Water and sanitation | 0.0051 (-0.0796 to 0.0898) | -0.4780 (-1.1727 to 0.2167) |
|  | Government and civil society | 0.0252 (-0.3067 to 0.3570) | 0.1567 (-2.5640 to 2.8774) |
|  | Conflict, peace and security | 0.0175 (-0.0773 to 0.1123) | -0.1803 (-0.9573 to 0.5966) |
|  | Other social services | 0.0484 (-0.0358 to 0.1325) | 0.9958 (0.3056 to 1.6861)* |
|  | Infrastructure | -0.0410 (-0.1700 to 0.0880) | -1.0660 (-2.1238 to -0.0083)* |
|  | Energy | 0.0155 (-0.1175 to 0.1485) | -0.4066 (-1.4971 to 0.6839) |
|  | Financial services and business support | 0.0978 (-0.0072 to 0.2029) | 0.0023 (-0.8589 to 0.8635) |
|  | Agriculture | 0.0695 (-0.0728 to 0.2119) | -0.1576 (-1.3247 to 1.0095) |
|  | Industry, construction and mining | -0.0313 (-0.1214 to 0.0588) | 0.3460 (-0.3930 to 1.0850) |
|  | Trade policy | -0.0163 (-0.0378 to 0.0053) | -0.0859 (-0.2626 to 0.0907) |
|  | Tourism | 0.0024 (-0.0039 to 0.0088) | -0.0250 (-0.0772 to 0.0272) |
|  | Environmental protection | -0.0712 (-0.2740 to 0.1315) | -0.3262 (-1.9883 to 1.3360) |
|  | Multisector | -0.1136 (-0.2904 to 0.0632) | -0.3525 (-1.8019 to 1.0969) |
|  | General budget support | -0.2151 (-0.2696 to -0.1606)*** | 2.1795 (1.7326 to 2.6264)*** |
|  | Food aid and commodity assistance | -0.1169 (-0.1740 to -0.0598)** | 0.2590 (-0.2089 to 0.7268) |
|  | Debt relief | -0.1186 (-0.6271 to 0.3900) | -0.3343 (-4.5039 to 3.8352) |
|  | Humanitarian aid | 0.5040 (0.3511 to 0.6568)*** | 0.1937 (-1.0595 to 1.4469) |
|  | Donor administration costs | -0.0537 (-0.1985 to 0.0912) | -0.0642 (-1.2519 to 1.1235) |
|  | Refugees in donor country | 0.1585 (-0.9204 to 1.2374) | -5.0265 (-13.8721 to 3.8192) |
|  | Unspecified | 0.1997 (0.0864 to 0.3130)** | -1.4854 (-2.4145 to -0.5564)** |

* p<0.05; ** p<0.01; *** p<0.001.

**Supplementary Table 8: Linear regression coefficients (95% confidence intervals) for the association between year and the COVID-19 pandemic with bilateral sector shares in G7 countries and other DAC countries**

| Donor | Sector | Year | COVID-19 |
| --- | --- | --- | --- |
| Canada | Education | -0.0642 (-0.3191 to 0.1906) | -0.5860 (-2.6756 to 1.5035) |
|  | Health | -0.1436 (-1.5396 to 1.2525) | 6.0781 (-5.3678 to 17.5240) |
|  | Water and sanitation | -0.0662 (-0.1951 to 0.0626) | -0.2664 (-1.3228 to 0.7901) |
|  | Government and civil society | -0.1153 (-0.7379 to 0.5072) | 3.7249 (-1.3793 to 8.8290) |
|  | Conflict, peace and security | -0.0265 (-0.2623 to 0.2094) | -0.1842 (-2.1179 to 1.7495) |
|  | Other social services | 0.0690 (-0.0293 to 0.1672) | -0.4591 (-1.2649 to 0.3466) |
|  | Infrastructure | -0.0659 (-0.1165 to -0.0152)* | 0.0914 (-0.3241 to 0.5070) |
|  | Energy | -0.1493 (-0.8520 to 0.5534) | 3.3684 (-2.3928 to 9.1296) |
|  | Financial services and business support | 0.0498 (-0.0840 to 0.1835) | -0.5610 (-1.6576 to 0.5355) |
|  | Agriculture | -0.3834 (-0.5841 to -0.1827)** | 2.8635 (1.2181 to 4.5088)** |
|  | Industry, construction and mining | 0.0580 (-0.1368 to 0.2527) | -1.3862 (-2.9832 to 0.2108) |
|  | Trade policy | -0.0753 (-0.1402 to -0.0105)* | 0.0019 (-0.5298 to 0.5336) |
|  | Tourism | -0.0031 (-0.0175 to 0.0112) | -0.0138 (-0.1312 to 0.1036) |
|  | Environmental protection | -0.0108 (-0.3584 to 0.3368) | -0.2846 (-3.1347 to 2.5655) |
|  | Multisector | -0.4157 (-1.3638 to 0.5324) | -2.3530 (-10.1265 to 5.4205) |
|  | General budget support | -0.1736 (-0.2531 to -0.0941)** | 0.4757 (-0.1763 to 1.1276) |
|  | Food aid and commodity assistance | -0.0740 (-0.1923 to 0.0442) | 0.0233 (-0.9462 to 0.9928) |
|  | Debt relief | NA | NA |
|  | Humanitarian aid | 0.8938 (0.0068 to 1.7809)* | -7.0460 (-14.3190 to 0.2268) |
|  | Donor administration costs | 0.0234 (-0.1904 to 0.2372) | -0.2190 (-1.9720 to 1.5339) |
|  | Refugees in donor country | 1.1759 (0.2786 to 2.0732)* | -4.0222 (-11.3790 to 3.3346) |
|  | Unspecified | -0.2635 (-0.4870 to -0.0399)* | 0.0090 (-1.8237 to 1.8418) |
| France | Education | -0.3832 (-0.6893 to -0.0770)* | -1.5264 (-4.0365 to 0.9837) |
|  | Health | 0.0272 (-0.5525 to 0.6069) | 2.9237 (-1.8292 to 7.6766) |
|  | Water and sanitation | 0.3825 (0.0942 to 0.6707)* | -3.2090 (-5.5722 to -0.8458)* |
|  | Government and civil society | 0.5599 (0.1323 to 0.9874)* | 1.5066 (-1.9990 to 5.0123) |
|  | Conflict, peace and security | -0.0735 (-0.1271 to -0.0200)* | 0.1225 (-0.3164 to 0.5615) |
|  | Other social services | -0.4216 (-0.9390 to 0.0959) | 3.1975 (-1.0449 to 7.4399) |
|  | Infrastructure | -0.0486 (-0.5106 to 0.4134) | -2.5762 (-6.3641 to 1.2117) |
|  | Energy | 0.7539 (0.3194 to 1.1884)** | -5.5534 (-9.1157 to -1.9910)** |
|  | Financial services and business support | 0.2889 (-0.1882 to 0.7661) | 3.2903 (-0.6216 to 7.2022) |
|  | Agriculture | 0.2626 (0.0545 to 0.4707)* | -0.4812 (-2.1871 to 1.2246) |
|  | Industry, construction and mining | 0.0336 (-0.1934 to 0.2607) | 2.0987 (0.2369 to 3.9605)* |
|  | Trade policy | 0.1164 (-0.0866 to 0.3193) | -0.1626 (-1.8269 to 1.5017) |
|  | Tourism | -0.0162 (-0.0418 to 0.0094) | -0.0126 (-0.2225 to 0.1972) |
|  | Environmental protection | -0.6017 (-1.1342 to -0.0692)* | 3.7896 (-0.5760 to 8.1552) |
|  | Multisector | -0.0489 (-1.0261 to 0.9282) | 0.9016 (-7.1100 to 8.9132) |
|  | General budget support | 0.0439 (-0.6331 to 0.7210) | -3.7984 (-9.3494 to 1.7526) |
|  | Food aid and commodity assistance | -0.0245 (-0.0637 to 0.0147) | -0.0206 (-0.3417 to 0.3006) |
|  | Debt relief | -2.0476 (-3.1345 to -0.9606)** | 7.3737 (-1.5384 to 16.2858) |
|  | Humanitarian aid | 0.1089 (-0.0235 to 0.2412) | -0.7527 (-1.8378 to 0.3323) |
|  | Donor administration costs | 0.2254 (0.0887 to 0.3621)** | -2.0354 (-3.1563 to -0.9144)** |
|  | Refugees in donor country | 0.6667 (0.2349 to 1.0986)** | -1.3738 (-4.9146 to 2.1671) |
|  | Unspecified | 0.1959 (-0.5917 to 0.9834) | -3.7020 (-10.1590 to 2.7550) |
| Germany | Education | -0.8401 (-1.4438 to -0.2365)* | 3.9306 (-1.0186 to 8.8798) |
|  | Health | -0.0705 (-0.2413 to 0.1003) | 5.6528 (4.2525 to 7.0530)*** |
|  | Water and sanitation | -0.3451 (-0.5089 to -0.1812)** | 0.1871 (-1.1564 to 1.5307) |
|  | Government and civil society | -0.5337 (-0.8744 to -0.1931)** | 3.6468 (0.8538 to 6.4398)* |
|  | Conflict, peace and security | 0.0024 (-0.1955 to 0.2003) | 0.4861 (-1.1362 to 2.1085) |
|  | Other social services | 0.0635 (-0.1063 to 0.2334) | 3.1962 (1.8037 to 4.5886)** |
|  | Infrastructure | -0.1103 (-0.3232 to 0.1025) | -0.3015 (-2.0468 to 1.4438) |
|  | Energy | -0.3824 (-0.8214 to 0.0565) | -1.0346 (-4.6337 to 2.5646) |
|  | Financial services and business support | -0.5845 (-1.2759 to 0.1068) | 1.9220 (-3.7462 to 7.5903) |
|  | Agriculture | -0.2320 (-0.4005 to -0.0634)* | 1.8376 (0.4553 to 3.2199)* |
|  | Industry, construction and mining | -0.0425 (-0.2123 to 0.1273) | 0.9962 (-0.3957 to 2.3882) |
|  | Trade policy | 0.0917 (-0.0290 to 0.2124) | -0.7521 (-1.7419 to 0.2378) |
|  | Tourism | -0.0063 (-0.0109 to -0.0017)* | 0.0265 (-0.0115 to 0.0645) |
|  | Environmental protection | -0.0894 (-0.2936 to 0.1147) | 0.3109 (-1.3629 to 1.9847) |
|  | Multisector | -0.0768 (-0.3535 to 0.1999) | 1.1970 (-1.0716 to 3.4656) |
|  | General budget support | 0.0272 (-0.1822 to 0.2366) | -0.7804 (-2.9021 to 1.3414) |
|  | Food aid and commodity assistance | 0.0844 (-0.0090 to 0.1778) | -0.0330 (-0.7987 to 0.7326) |
|  | Debt relief | -0.9736 (-1.6162 to -0.3311)** | 2.3870 (-2.8812 to 7.6552) |
|  | Humanitarian aid | 1.0852 (0.6527 to 1.5177)*** | -4.4282 (-7.9744 to -0.8821)* |
|  | Donor administration costs | -0.0980 (-0.3365 to 0.1406) | 0.6607 (-1.2951 to 2.6164) |
|  | Refugees in donor country | 3.0298 (0.5450 to 5.5146)* | -19.0357 (-39.4084 to 1.3371) |
|  | Unspecified | 0.0014 (-0.0285 to 0.0314) | -0.0869 (-0.3323 to 0.1586) |
| Italy | Education | 0.1185 (-0.4721 to 0.7090) | 2.7051 (-2.1365 to 7.5467) |
|  | Health | 0.1011 (-0.4670 to 0.6693) | 5.6699 (1.0118 to 10.3280)* |
|  | Water and sanitation | -0.0549 (-0.2223 to 0.1126) | 0.7805 (-0.5924 to 2.1534) |
|  | Government and civil society | 0.4780 (0.0440 to 0.9121)* | -2.9007 (-6.4593 to 0.6578) |
|  | Conflict, peace and security | 0.0491 (-0.0234 to 0.1216) | -0.3240 (-0.9185 to 0.2705) |
|  | Other social services | -0.0039 (-0.2449 to 0.2371) | 0.0247 (-1.9512 to 2.0006) |
|  | Infrastructure | -0.2619 (-0.5787 to 0.0550) | 3.1499 (0.5521 to 5.7476)* |
|  | Energy | 0.0920 (-0.3075 to 0.4916) | 1.7928 (-1.4833 to 5.0688) |
|  | Financial services and business support | 0.0241 (-0.0278 to 0.0759) | -0.2195 (-0.6446 to 0.2057) |
|  | Agriculture | -0.0477 (-0.5992 to 0.5039) | 1.8949 (-2.6274 to 6.4173) |
|  | Industry, construction and mining | -0.2438 (-0.6824 to 0.1949) | 2.7768 (-0.8193 to 6.3730) |
|  | Trade policy | 0.0065 (-0.0419 to 0.0550) | 0.2926 (-0.1044 to 0.6897) |
|  | Tourism | 0.0008 (-0.0238 to 0.0255) | -0.0403 (-0.2426 to 0.1619) |
|  | Environmental protection | 0.0056 (-0.2867 to 0.2979) | -0.4317 (-2.8283 to 1.9650) |
|  | Multisector | 0.3792 (-0.1635 to 0.9219) | 1.5532 (-2.8962 to 6.0025) |
|  | General budget support | NA | NA |
|  | Food aid and commodity assistance | -0.0910 (-0.2256 to 0.0436) | 0.0086 (-1.0953 to 1.1125) |
|  | Debt relief | -2.2339 (-5.7901 to 1.3223) | 19.0454 (-10.1117 to 48.2025) |
|  | Humanitarian aid | 0.3933 (-0.4359 to 1.2224) | 0.4829 (-6.3153 to 7.2811) |
|  | Donor administration costs | -0.2045 (-0.5583 to 0.1493) | 1.1189 (-1.7821 to 4.0199) |
|  | Refugees in donor country | 1.7255 (-2.1200 to 5.5709) | -37.1056 (-68.6341 to -5.5771)* |
|  | Unspecified | -0.1386 (-0.4431 to 0.1660) | -0.4835 (-2.9804 to 2.0133) |
| Japan | Education | -0.2573 (-0.5922 to 0.0776) | 0.2409 (-2.5051 to 2.9870) |
|  | Health | 0.0361 (-0.1852 to 0.2574) | 4.7640 (2.9499 to 6.5781)*** |
|  | Water and sanitation | -0.4411 (-0.8362 to -0.0461)* | -2.4215 (-5.6606 to 0.8176) |
|  | Government and civil society | -0.1025 (-0.2154 to 0.0105) | -0.8783 (-1.8045 to 0.0478) |
|  | Conflict, peace and security | -0.0165 (-0.0532 to 0.0201) | 0.1813 (-0.1195 to 0.4820) |
|  | Other social services | 0.0446 (-0.1792 to 0.2684) | 0.4220 (-1.4129 to 2.2570) |
|  | Infrastructure | 1.9616 (1.0982 to 2.8250)** | -8.2797 (-15.3587 to -1.2007)* |
|  | Energy | 0.5627 (-0.1668 to 1.2923) | -4.4642 (-10.4457 to 1.5173) |
|  | Financial services and business support | -0.0088 (-0.0389 to 0.0213) | 0.0415 (-0.2051 to 0.2881) |
|  | Agriculture | -0.0533 (-0.2933 to 0.1867) | -0.7571 (-2.7250 to 1.2108) |
|  | Industry, construction and mining | -0.1824 (-0.4874 to 0.1225) | 1.5159 (-0.9844 to 4.0162) |
|  | Trade policy | -0.0537 (-0.0987 to -0.0087)* | -0.0045 (-0.3738 to 0.3647) |
|  | Tourism | -0.0245 (-0.0886 to 0.0396) | -0.1968 (-0.7223 to 0.3287) |
|  | Environmental protection | -0.1887 (-0.2853 to -0.0922)** | 0.0277 (-0.7638 to 0.8191) |
|  | Multisector | 0.5009 (0.1086 to 0.8933)* | 0.3464 (-2.8705 to 3.5632) |
|  | General budget support | -0.1149 (-1.3267 to 1.0968) | 7.2081 (-2.7268 to 17.1429) |
|  | Food aid and commodity assistance | -0.2359 (-0.3797 to -0.0920)** | 0.0128 (-1.1666 to 1.1922) |
|  | Debt relief | NA | NA |
|  | Humanitarian aid | -0.1515 (-0.6837 to 0.3808) | -0.3708 (-4.7350 to 3.9933) |
|  | Donor administration costs | 0.0380 (-0.1569 to 0.2328) | -0.7647 (-2.3621 to 0.8328) |
|  | Refugees in donor country | -0.0004 (-0.0006 to -0.0001)** | 0.0007 (-0.0014 to 0.0027) |
|  | Unspecified | -0.0405 (-0.2487 to 0.1677) | -0.2238 (-1.9308 to 1.4833) |
| United Kingdom | Education | -0.6476 (-1.0152 to -0.2801)** | -0.4819 (-3.4957 to 2.5319) |
|  | Health | -0.6966 (-1.2239 to -0.1692)* | 3.2960 (-1.0274 to 7.6195) |
|  | Water and sanitation | -0.0163 (-0.0908 to 0.0581) | -0.8668 (-1.4774 to -0.2563)* |
|  | Government and civil society | -0.3023 (-0.4517 to -0.1529)** | -0.0857 (-1.3105 to 1.1391) |
|  | Conflict, peace and security | 0.2386 (0.0249 to 0.4523)* | -1.5440 (-3.2960 to 0.2081) |
|  | Other social services | -0.2396 (-0.4729 to -0.0063)* | 0.2068 (-1.7057 to 2.1193) |
|  | Infrastructure | -0.2560 (-0.3907 to -0.1213)** | 0.3153 (-0.7890 to 1.4195) |
|  | Energy | -0.0224 (-0.3076 to 0.2629) | 0.6902 (-1.6485 to 3.0290) |
|  | Financial services and business support | 0.5683 (0.1484 to 0.9883)* | -2.2878 (-5.7308 to 1.1553) |
|  | Agriculture | -0.0827 (-0.2761 to 0.1106) | -1.2437 (-2.8293 to 0.3418) |
|  | Industry, construction and mining | 0.3417 (0.1099 to 0.5736)** | -0.4902 (-2.3911 to 1.4107) |
|  | Trade policy | -0.0253 (-0.0730 to 0.0223) | 0.1082 (-0.2825 to 0.4989) |
|  | Tourism | -0.0020 (-0.0046 to 0.0007) | 0.0026 (-0.0193 to 0.0244) |
|  | Environmental protection | -0.0540 (-0.3372 to 0.2292) | -0.8437 (-3.1659 to 1.4785) |
|  | Multisector | 0.4369 (0.2486 to 0.6253)** | -2.1383 (-3.6830 to -0.5937)* |
|  | General budget support | NA | NA |
|  | Food aid and commodity assistance | -0.1411 (-0.2511 to -0.0312)* | 0.4909 (-0.4105 to 1.3922) |
|  | Debt relief | -0.2645 (-0.4420 to -0.0870)* | 2.8808 (1.3057 to 4.4559)** |
|  | Humanitarian aid | 0.8419 (0.0046 to 1.6792)* | -4.7217 (-11.5867 to 2.1433) |
|  | Donor administration costs | 0.3213 (-0.0101 to 0.6526) | 1.9535 (-0.7635 to 4.6704) |
|  | Refugees in donor country | 0.6845 (0.0978 to 1.2713)* | 4.0799 (-0.7310 to 8.8909) |
|  | Unspecified | -0.1113 (-0.4345 to 0.2119) | -0.1245 (-2.7744 to 2.5254) |
| United States | Education | 0.2735 (0.1138 to 0.4332)** | -1.8754 (-3.1849 to -0.5660)* |
|  | Health | -0.1615 (-0.8557 to 0.5326) | -2.5437 (-8.2349 to 3.1474) |
|  | Water and sanitation | -0.0122 (-0.0735 to 0.0491) | -0.2821 (-0.7847 to 0.2205) |
|  | Government and civil society | -0.4386 (-1.0281 to 0.1508) | -1.1556 (-5.9886 to 3.6774) |
|  | Conflict, peace and security | 0.0519 (-0.0213 to 0.1252) | -0.4335 (-1.0342 to 0.1672) |
|  | Other social services | -0.2141 (-0.3327 to -0.0955)** | 0.5691 (-0.4036 to 1.5418) |
|  | Infrastructure | -0.4478 (-0.5502 to -0.3454)*** | 1.0796 (0.2399 to 1.9193)* |
|  | Energy | -0.0492 (-0.1091 to 0.0106) | 0.2702 (-0.2204 to 0.7608) |
|  | Financial services and business support | -0.1330 (-0.2250 to -0.0410)* | 0.3613 (-0.3928 to 1.1154) |
|  | Agriculture | -0.2083 (-0.3360 to -0.0806)** | -0.5658 (-1.6128 to 0.4812) |
|  | Industry, construction and mining | -0.0422 (-0.0590 to -0.0254)*** | -0.0432 (-0.1809 to 0.0946) |
|  | Trade policy | -0.0645 (-0.1091 to -0.0199)* | 0.0122 (-0.3534 to 0.3779) |
|  | Tourism | -0.0066 (-0.0184 to 0.0052) | 0.0076 (-0.1056 to 0.1207) |
|  | Environmental protection | 0.0154 (-0.0818 to 0.1126) | -0.7648 (-1.5615 to 0.0319) |
|  | Multisector | -0.1117 (-0.2739 to 0.0505) | 0.2805 (-1.0495 to 1.6105) |
|  | General budget support | -0.1537 (-0.3079 to 0.0005) | 0.6378 (-0.6263 to 1.9020) |
|  | Food aid and commodity assistance | 0.0267 (-0.1311 to 0.1846) | -0.2980 (-1.5922 to 0.9963) |
|  | Debt relief | -0.4373 (-0.8692 to -0.0054)* | 2.0065 (-1.5347 to 5.5477) |
|  | Humanitarian aid | 1.4283 (1.0001 to 1.8565)*** | 1.2927 (-2.2182 to 4.8036) |
|  | Donor administration costs | 0.3061 (0.0762 to 0.5360)* | -0.4578 (-2.3426 to 1.4270) |
|  | Refugees in donor country | 0.4499 (-0.0963 to 0.9962) | 1.3659 (-3.1128 to 5.8447) |
|  | Unspecified | -0.0711 (-0.1563 to 0.0142) | 0.5339 (-0.1652 to 1.2329) |
| Others | Education | -0.0687 (-0.3064 to 0.1690) | 0.2506 (-1.6982 to 2.1994) |
|  | Health | 0.0593 (-0.3293 to 0.4478) | 6.4781 (3.2921 to 9.6641)** |
|  | Water and sanitation | -0.0279 (-0.1181 to 0.0623) | -0.3459 (-1.0858 to 0.3939) |
|  | Government and civil society | -0.0520 (-0.4475 to 0.3435) | 0.6778 (-2.5649 to 3.9204) |
|  | Conflict, peace and security | 0.0016 (-0.1111 to 0.1143) | -0.1935 (-1.1177 to 0.7308) |
|  | Other social services | 0.0170 (-0.0917 to 0.1256) | 1.0152 (0.1246 to 1.9057)* |
|  | Infrastructure | -0.0674 (-0.1590 to 0.0242) | -0.2691 (-1.0201 to 0.4820) |
|  | Energy | -0.0495 (-0.1898 to 0.0909) | 0.0297 (-1.1208 to 1.1802) |
|  | Financial services and business support | 0.1170 (0.0195 to 0.2146)* | -0.1482 (-0.9481 to 0.6517) |
|  | Agriculture | -0.0135 (-0.1444 to 0.1175) | -0.2893 (-1.3631 to 0.7846) |
|  | Industry, construction and mining | -0.0074 (-0.0719 to 0.0572) | 0.1486 (-0.3807 to 0.6779) |
|  | Trade policy | -0.0206 (-0.0470 to 0.0059) | -0.0809 (-0.2977 to 0.1359) |
|  | Tourism | -0.0008 (-0.0101 to 0.0085) | -0.0117 (-0.0880 to 0.0647) |
|  | Environmental protection | -0.0808 (-0.3182 to 0.1566) | 0.1352 (-1.8112 to 2.0817) |
|  | Multisector | -0.1649 (-0.3969 to 0.0671) | -0.2494 (-2.1516 to 1.6527) |
|  | General budget support | -0.2316 (-0.3112 to -0.1520)*** | 0.9889 (0.3361 to 1.6417)** |
|  | Food aid and commodity assistance | -0.1465 (-0.2193 to -0.0737)** | 0.4301 (-0.1666 to 1.0268) |
|  | Debt relief | -0.1465 (-0.8784 to 0.5854) | -0.9819 (-6.9826 to 5.0189) |
|  | Humanitarian aid | 0.4591 (0.2430 to 0.6751)** | 0.3536 (-1.4177 to 2.1248) |
|  | Donor administration costs | 0.0332 (-0.1473 to 0.2136) | 0.1507 (-1.3289 to 1.6304) |
|  | Refugees in donor country | 0.2936 (-1.1540 to 1.7412) | -7.1108 (-18.9793 to 4.7577) |
|  | Unspecified | 0.0972 (-0.0281 to 0.2225) | -0.9779 (-2.0051 to 0.0493) |

* p<0.05; ** p<0.01; *** p<0.001. NA: The regression model could not be constructed due to a small number of contribution years

**Supplementary Table 9: Linear regression coefficients (95% confidence intervals) for the association between year and the COVID-19 pandemic with multilateral sector shares in G7 countries and other DAC countries**

| Donor | Sector | Year | COVID-19 |
| --- | --- | --- | --- |
| Canada | Education | 0.1275 (-0.6554 to 0.9105) | -1.4430 (-7.8623 to 4.9763) |
|  | Health | -3.4354 (-10.3056 to 3.4348) | 19.6537 (-36.6742 to 75.9816) |
|  | Water and sanitation | 0.0829 (-0.1848 to 0.3506) | -0.3368 (-2.5319 to 1.8582) |
|  | Government and civil society | 0.1503 (-1.5271 to 1.8278) | -4.0547 (-17.8080 to 9.6986) |
|  | Conflict, peace and security | 0.0832 (-0.1104 to 0.2768) | -0.4129 (-2.0000 to 1.1742) |
|  | Other social services | 0.7065 (-0.1289 to 1.5419) | -3.9685 (-10.8178 to 2.8808) |
|  | Infrastructure | 0.0646 (-0.3047 to 0.4339) | -1.3462 (-4.3743 to 1.6820) |
|  | Energy | 0.1638 (-0.1800 to 0.5077) | -1.4230 (-4.2426 to 1.3965) |
|  | Financial services and business support | 0.0387 (-0.1461 to 0.2235) | -0.0050 (-1.5205 to 1.5104) |
|  | Agriculture | 0.0848 (-0.4729 to 0.6425) | 1.9988 (-2.5736 to 6.5712) |
|  | Industry, construction and mining | 0.0277 (-0.0686 to 0.1240) | -0.1038 (-0.8931 to 0.6854) |
|  | Trade policy | 0.0218 (-0.0221 to 0.0656) | 0.0588 (-0.3005 to 0.4181) |
|  | Tourism | 0.0189 (0.0052 to 0.0325)* | -0.1400 (-0.2518 to -0.0282)* |
|  | Environmental protection | -0.2075 (-0.7969 to 0.3819) | -0.9215 (-5.7539 to 3.9109) |
|  | Multisector | -0.2819 (-0.8519 to 0.2881) | -0.3232 (-4.9965 to 4.3501) |
|  | General budget support | 0.0200 (-0.0445 to 0.0845) | 0.3544 (-0.1745 to 0.8834) |
|  | Food aid and commodity assistance | -0.0009 (-0.0543 to 0.0525) | 0.1859 (-0.2521 to 0.6239) |
|  | Debt relief | 0.0064 (-0.0131 to 0.0259) | -0.0834 (-0.2433 to 0.0765) |
|  | Humanitarian aid | 1.2316 (-0.2767 to 2.7399) | -1.2279 (-13.5945 to 11.1387) |
|  | Donor administration costs | -0.0780 (-0.7552 to 0.5993) | -0.8497 (-6.4027 to 4.7033) |
|  | Refugees in donor country | NA | NA |
|  | Unspecified | 1.1750 (-0.0132 to 2.3633) | -5.6122 (-15.3547 to 4.1302) |
| France | Education | 0.0670 (-0.1568 to 0.2908) | -1.3314 (-3.1662 to 0.5034) |
|  | Health | 0.0004 (-0.8418 to 0.8427) | -2.2934 (-9.1986 to 4.6118) |
|  | Water and sanitation | 0.0524 (-0.2406 to 0.3453) | -0.7908 (-3.1925 to 1.6108) |
|  | Government and civil society | 0.5451 (-0.3595 to 1.4497) | -3.4336 (-10.8505 to 3.9832) |
|  | Conflict, peace and security | 0.1192 (-0.0486 to 0.2869) | -0.7528 (-2.1284 to 0.6228) |
|  | Other social services | -0.0113 (-0.3198 to 0.2971) | 0.5880 (-1.9410 to 3.1171) |
|  | Infrastructure | -0.4024 (-0.8117 to 0.0069) | -2.6383 (-5.9942 to 0.7175) |
|  | Energy | -0.0603 (-0.5597 to 0.4392) | -1.8465 (-5.9417 to 2.2486) |
|  | Financial services and business support | 0.0279 (-0.3031 to 0.3588) | -1.1303 (-3.8439 to 1.5833) |
|  | Agriculture | 0.2843 (-0.3624 to 0.9310) | 1.5540 (-3.7481 to 6.8562) |
|  | Industry, construction and mining | -0.2335 (-0.5684 to 0.1015) | 1.3295 (-1.4167 to 4.0758) |
|  | Trade policy | -0.0248 (-0.0462 to -0.0035)* | -0.1026 (-0.2774 to 0.0723) |
|  | Tourism | 0.0037 (-0.0046 to 0.0120) | -0.0241 (-0.0924 to 0.0441) |
|  | Environmental protection | 0.1482 (-0.2477 to 0.5442) | -2.1517 (-5.3984 to 1.0950) |
|  | Multisector | 0.3424 (0.0824 to 0.6023)* | -0.3755 (-2.5065 to 1.7556) |
|  | General budget support | -1.1493 (-1.8624 to -0.4362)** | 15.3983 (9.5517 to 21.2449)*** |
|  | Food aid and commodity assistance | -0.0498 (-0.1127 to 0.0130) | -0.1986 (-0.7141 to 0.3168) |
|  | Debt relief | -0.0898 (-0.1382 to -0.0413)** | 1.4613 (1.0641 to 1.8585)*** |
|  | Humanitarian aid | 0.4733 (0.0174 to 0.9292)* | -2.2005 (-5.9383 to 1.5372) |
|  | Donor administration costs | -0.0880 (-0.3237 to 0.1477) | -0.4859 (-2.4183 to 1.4465) |
|  | Refugees in donor country | NA | NA |
|  | Unspecified | 0.0486 (-0.0598 to 0.1570) | -0.5783 (-1.4671 to 0.3106) |
| Germany | Education | 0.0763 (-0.1267 to 0.2793) | -1.0166 (-2.6812 to 0.6480) |
|  | Health | 0.1959 (-0.8480 to 1.2398) | 11.2002 (2.6414 to 19.7590)* |
|  | Water and sanitation | -0.0024 (-0.1028 to 0.0979) | -1.3037 (-2.1267 to -0.4807)** |
|  | Government and civil society | 0.4515 (-0.0150 to 0.9179) | -1.9304 (-5.7546 to 1.8939) |
|  | Conflict, peace and security | 0.1132 (-0.0096 to 0.2360) | -0.7773 (-1.7841 to 0.2295) |
|  | Other social services | -0.0825 (-0.3286 to 0.1636) | 1.3549 (-0.6631 to 3.3730) |
|  | Infrastructure | -0.4571 (-0.7850 to -0.1292)* | -3.5150 (-6.2034 to -0.8266)* |
|  | Energy | -0.2594 (-0.5870 to 0.0683) | -1.3574 (-4.0439 to 1.3291) |
|  | Financial services and business support | -0.1749 (-0.7058 to 0.3561) | -0.8364 (-5.1894 to 3.5166) |
|  | Agriculture | 0.2191 (-0.0575 to 0.4957) | -3.1673 (-5.4347 to -0.8998)* |
|  | Industry, construction and mining | -0.3108 (-0.7259 to 0.1043) | 1.1080 (-2.2954 to 4.5115) |
|  | Trade policy | -0.0447 (-0.0830 to -0.0064)* | -0.1861 (-0.5002 to 0.1279) |
|  | Tourism | 0.0049 (-0.0069 to 0.0167) | -0.0675 (-0.1645 to 0.0295) |
|  | Environmental protection | -0.0702 (-0.3921 to 0.2518) | -0.4698 (-3.1094 to 2.1699) |
|  | Multisector | 0.3758 (0.1380 to 0.6137)** | -2.9817 (-4.9314 to -1.0320)** |
|  | General budget support | -0.3958 (-0.6597 to -0.1320)** | 5.3429 (3.1797 to 7.5061)*** |
|  | Food aid and commodity assistance | -0.0606 (-0.1621 to 0.0408) | -0.4344 (-1.2664 to 0.3977) |
|  | Debt relief | -0.0262 (-0.0800 to 0.0276) | 0.2869 (-0.1541 to 0.7278) |
|  | Humanitarian aid | 0.3716 (0.0994 to 0.6438)* | -0.3074 (-2.5395 to 1.9246) |
|  | Donor administration costs | -0.0343 (-0.1029 to 0.0342) | -0.5600 (-1.1218 to 0.0018) |
|  | Refugees in donor country | NA | NA |
|  | Unspecified | 0.1155 (-0.0517 to 0.2826) | -0.3856 (-1.7562 to 0.9851) |
| Italy | Education | 0.0802 (-0.1080 to 0.2685) | -1.0629 (-2.6063 to 0.4805) |
|  | Health | 0.4035 (-0.6589 to 1.4660) | 6.7561 (-1.9548 to 15.4669) |
|  | Water and sanitation | -0.0062 (-0.1296 to 0.1171) | -0.8955 (-1.9068 to 0.1158) |
|  | Government and civil society | 0.5662 (0.0598 to 1.0726)* | -2.8846 (-7.0363 to 1.2670) |
|  | Conflict, peace and security | 0.1060 (-0.0532 to 0.2652) | -0.6633 (-1.9686 to 0.6420) |
|  | Other social services | -0.1266 (-0.3759 to 0.1228) | 1.1633 (-0.8812 to 3.2078) |
|  | Infrastructure | -0.4852 (-0.8518 to -0.1186)* | -3.0017 (-6.0075 to 0.0041) |
|  | Energy | -0.2845 (-0.6365 to 0.0676) | -0.7733 (-3.6598 to 2.1131) |
|  | Financial services and business support | -0.1609 (-0.6712 to 0.3493) | -0.2965 (-4.4799 to 3.8870) |
|  | Agriculture | 0.3122 (0.0271 to 0.5973)* | -2.1517 (-4.4889 to 0.1855) |
|  | Industry, construction and mining | -0.3567 (-0.8093 to 0.0959) | 1.6046 (-2.1063 to 5.3155) |
|  | Trade policy | -0.0524 (-0.0978 to -0.0070)* | -0.1031 (-0.4754 to 0.2692) |
|  | Tourism | 0.0055 (-0.0066 to 0.0176) | -0.0650 (-0.1643 to 0.0344) |
|  | Environmental protection | 0.0551 (-0.0553 to 0.1655) | -0.6923 (-1.5973 to 0.2128) |
|  | Multisector | 0.3823 (0.0752 to 0.6894)* | -2.2270 (-4.7446 to 0.2907) |
|  | General budget support | -0.4446 (-0.7672 to -0.1221)* | 6.9085 (4.2640 to 9.5530)*** |
|  | Food aid and commodity assistance | -0.0822 (-0.1719 to 0.0075) | -0.1614 (-0.8971 to 0.5742) |
|  | Debt relief | -0.0357 (-0.0930 to 0.0216) | 0.4166 (-0.0529 to 0.8861) |
|  | Humanitarian aid | 0.2900 (0.0583 to 0.5217)* | -1.2049 (-3.1047 to 0.6949) |
|  | Donor administration costs | -0.0809 (-0.1841 to 0.0223) | -0.7206 (-1.5665 to 0.1254) |
|  | Refugees in donor country | NA | NA |
|  | Unspecified | -0.0800 (-0.2316 to 0.0715) | 0.0506 (-1.1918 to 1.2931) |
| Japan | Education | -0.1891 (-0.7838 to 0.4056) | -0.2882 (-5.1640 to 4.5877) |
|  | Health | -0.5405 (-2.8583 to 1.7773) | 4.2223 (-14.7812 to 23.2258) |
|  | Water and sanitation | 0.1678 (-0.3754 to 0.7110) | -0.8215 (-5.2754 to 3.6325) |
|  | Government and civil society | 0.4209 (-2.0363 to 2.8782) | -5.9826 (-26.1292 to 14.1641) |
|  | Conflict, peace and security | -0.0143 (-0.0444 to 0.0158) | 0.1543 (-0.0924 to 0.4010) |
|  | Other social services | 0.2326 (-0.2683 to 0.7334) | 1.0276 (-3.0789 to 5.1342) |
|  | Infrastructure | -0.3268 (-1.3618 to 0.7082) | -0.5341 (-9.0200 to 7.9518) |
|  | Energy | 0.4242 (-0.4936 to 1.3420) | -1.4135 (-8.9384 to 6.1115) |
|  | Financial services and business support | 0.0656 (-0.3995 to 0.5307) | -0.2509 (-4.0644 to 3.5626) |
|  | Agriculture | 0.2135 (-0.6838 to 1.1107) | 2.5273 (-4.8293 to 9.8839) |
|  | Industry, construction and mining | 0.0202 (-0.1907 to 0.2311) | -0.0508 (-1.7799 to 1.6784) |
|  | Trade policy | 0.0312 (-0.0789 to 0.1412) | 0.0248 (-0.8775 to 0.9271) |
|  | Tourism | 0.0168 (-0.0072 to 0.0408) | -0.0397 (-0.2364 to 0.1570) |
|  | Environmental protection | -0.3954 (-1.3855 to 0.5947) | -0.0319 (-8.1496 to 8.0859) |
|  | Multisector | -0.1618 (-0.3468 to 0.0231) | 3.2988 (1.7823 to 4.8153)** |
|  | General budget support | -0.3404 (-0.7846 to 0.1037) | 1.1902 (-2.4513 to 4.8318) |
|  | Food aid and commodity assistance | -0.0159 (-0.0306 to -0.0013)* | -0.0250 (-0.1452 to 0.0953) |
|  | Debt relief | -0.1796 (-0.3716 to 0.0124) | 1.6061 (0.0317 to 3.1805)* |
|  | Humanitarian aid | 0.5708 (-0.1994 to 1.3410) | -2.4110 (-8.7259 to 3.9039) |
|  | Donor administration costs | -0.5248 (-0.8511 to -0.1985)** | 0.6235 (-2.0517 to 3.2986) |
|  | Refugees in donor country | NA | NA |
|  | Unspecified | 0.5252 (-0.0848 to 1.1353) | -2.8258 (-7.8277 to 2.1760) |
| United Kingdom | Education | 0.1145 (-0.1193 to 0.3483) | -2.2759 (-4.1928 to -0.3590)* |
|  | Health | -1.1002 (-2.8331 to 0.6327) | 21.4989 (7.2914 to 35.7064)** |
|  | Water and sanitation | -0.0227 (-0.2393 to 0.1940) | -1.6169 (-3.3933 to 0.1595) |
|  | Government and civil society | 0.3115 (-0.3276 to 0.9506) | -2.9457 (-8.1855 to 2.2942) |
|  | Conflict, peace and security | 0.0714 (-0.2167 to 0.3595) | -0.5517 (-2.9140 to 1.8107) |
|  | Other social services | 0.0076 (-0.2597 to 0.2750) | -0.8397 (-3.0316 to 1.3521) |
|  | Infrastructure | -0.4390 (-1.0005 to 0.1226) | -3.2297 (-7.8339 to 1.3746) |
|  | Energy | -0.2747 (-0.6829 to 0.1335) | -1.7218 (-5.0687 to 1.6252) |
|  | Financial services and business support | -0.1866 (-0.7059 to 0.3328) | -0.3544 (-4.6124 to 3.9037) |
|  | Agriculture | 0.2427 (-0.1898 to 0.6751) | -3.7143 (-7.2599 to -0.1688)* |
|  | Industry, construction and mining | -0.2774 (-0.6016 to 0.0469) | 0.9833 (-1.6750 to 3.6416) |
|  | Trade policy | -0.0414 (-0.1056 to 0.0228) | -0.1507 (-0.6771 to 0.3758) |
|  | Tourism | 0.0033 (-0.0025 to 0.0092) | -0.0630 (-0.1111 to -0.0149)* |
|  | Environmental protection | -0.1170 (-0.4865 to 0.2525) | -0.6485 (-3.6779 to 2.3810) |
|  | Multisector | 0.1066 (-0.2684 to 0.4815) | -2.0287 (-5.1028 to 1.0455) |
|  | General budget support | 1.0184 (-1.5515 to 3.5882) | 0.1256 (-20.9444 to 21.1955) |
|  | Food aid and commodity assistance | -0.0646 (-0.1688 to 0.0397) | -0.1128 (-0.9675 to 0.7418) |
|  | Debt relief | -0.0311 (-0.1634 to 0.1012) | 1.5301 (0.4452 to 2.6150)* |
|  | Humanitarian aid | 0.5479 (-0.3170 to 1.4127) | -1.4541 (-8.5447 to 5.6364) |
|  | Donor administration costs | -0.1214 (-0.5305 to 0.2878) | -0.7226 (-4.0771 to 2.6320) |
|  | Refugees in donor country | NA | NA |
|  | Unspecified | 0.2562 (-0.1908 to 0.7033) | -1.7118 (-5.3771 to 1.9535) |
| United States | Education | -0.0483 (-0.2376 to 0.1409) | -0.6183 (-2.1700 to 0.9334) |
|  | Health | -1.4007 (-3.4238 to 0.6224) | 16.5390 (-0.0482 to 33.1261) |
|  | Water and sanitation | 0.0382 (-0.0490 to 0.1254) | -0.4745 (-1.1895 to 0.2405) |
|  | Government and civil society | 0.1513 (-1.0620 to 1.3647) | -3.8142 (-13.7622 to 6.1338) |
|  | Conflict, peace and security | 0.0186 (-0.0572 to 0.0945) | -0.1350 (-0.7568 to 0.4868) |
|  | Other social services | 0.0361 (-0.3894 to 0.4615) | 1.2576 (-2.2305 to 4.7458) |
|  | Infrastructure | 0.0154 (-0.0457 to 0.0765) | -0.3574 (-0.8584 to 0.1437) |
|  | Energy | 0.0295 (-0.0737 to 0.1327) | -0.4696 (-1.3156 to 0.3763) |
|  | Financial services and business support | -0.0225 (-0.0382 to -0.0067)* | 0.1823 (0.0530 to 0.3116)* |
|  | Agriculture | 0.2638 (-0.3730 to 0.9007) | -2.6155 (-7.8372 to 2.6062) |
|  | Industry, construction and mining | 0.0050 (-0.0067 to 0.0167) | -0.0524 (-0.1484 to 0.0437) |
|  | Trade policy | 0.0082 (-0.0012 to 0.0176) | -0.0076 (-0.0846 to 0.0694) |
|  | Tourism | 0.0067 (0.0024 to 0.0109)** | -0.0506 (-0.0854 to -0.0157)* |
|  | Environmental protection | -0.1577 (-0.3926 to 0.0772) | -0.8969 (-2.8227 to 1.0290) |
|  | Multisector | -0.4316 (-0.6636 to -0.1996)** | -0.3111 (-2.2133 to 1.5911) |
|  | General budget support | 0.0131 (0.0005 to 0.0257)* | 0.0466 (-0.0569 to 0.1500) |
|  | Food aid and commodity assistance | -0.0093 (-0.0227 to 0.0041) | -0.0082 (-0.1183 to 0.1018) |
|  | Debt relief | 0.0063 (-0.0056 to 0.0182) | -0.0512 (-0.1488 to 0.0464) |
|  | Humanitarian aid | 0.7848 (0.0650 to 1.5046)* | -1.9012 (-7.8029 to 4.0005) |
|  | Donor administration costs | -0.1071 (-0.4111 to 0.1968) | -1.3555 (-3.8475 to 1.1364) |
|  | Refugees in donor country | NA | NA |
|  | Unspecified | 0.8002 (0.3743 to 1.2262)** | -4.9063 (-8.3986 to -1.4140)* |
| Others | Education | 0.0252 (-0.1594 to 0.2099) | -0.6357 (-2.1495 to 0.8782) |
|  | Health | -1.3879 (-2.5145 to -0.2614)* | 4.9274 (-4.3091 to 14.1638) |
|  | Water and sanitation | 0.0976 (-0.0311 to 0.2264) | -0.8089 (-1.8645 to 0.2468) |
|  | Government and civil society | 0.2867 (-0.2303 to 0.8036) | -1.1963 (-5.4344 to 3.0418) |
|  | Conflict, peace and security | 0.0835 (-0.0131 to 0.1801) | -0.1950 (-0.9869 to 0.5969) |
|  | Other social services | 0.0789 (-0.1817 to 0.3395) | 0.8601 (-1.2764 to 2.9965) |
|  | Infrastructure | -0.0813 (-0.4372 to 0.2747) | -2.7396 (-5.6582 to 0.1790) |
|  | Energy | 0.1181 (-0.1071 to 0.3433) | -1.3995 (-3.2458 to 0.4468) |
|  | Financial services and business support | 0.0214 (-0.2830 to 0.3258) | 0.2701 (-2.2256 to 2.7657) |
|  | Agriculture | 0.2835 (-0.0592 to 0.6262) | -0.0168 (-2.8263 to 2.7928) |
|  | Industry, construction and mining | -0.1084 (-0.3272 to 0.1104) | 0.8432 (-0.9506 to 2.6370) |
|  | Trade policy | -0.0027 (-0.0376 to 0.0322) | -0.0977 (-0.3837 to 0.1883) |
|  | Tourism | 0.0105 (0.0041 to 0.0169)** | -0.0577 (-0.1101 to -0.0052)* |
|  | Environmental protection | -0.0345 (-0.2755 to 0.2066) | -1.3518 (-3.3281 to 0.6246) |
|  | Multisector | 0.0270 (-0.1493 to 0.2033) | -0.6151 (-2.0604 to 0.8302) |
|  | General budget support | -0.1941 (-0.3915 to 0.0033) | 4.6289 (3.0103 to 6.2474)*** |
|  | Food aid and commodity assistance | -0.0384 (-0.0865 to 0.0098) | -0.1594 (-0.5545 to 0.2357) |
|  | Debt relief | -0.0252 (-0.2833 to 0.2329) | 1.0041 (-1.1122 to 3.1204) |
|  | Humanitarian aid | 0.6103 (0.3937 to 0.8268)*** | -0.1116 (-1.8870 to 1.6639) |
|  | Donor administration costs | -0.2200 (-0.3950 to -0.0451)* | -0.4350 (-1.8694 to 0.9993) |
|  | Refugees in donor country | NA | NA |
|  | Unspecified | 0.4527 (0.1529 to 0.7525)** | -2.7162 (-5.1743 to -0.2581)* |

* p<0.05; ** p<0.01; *** p<0.001. NA: The regression model could not be constructed due to a small number of contribution years
